# scRiskCell: A single‐cell framework for quantifying islet risk cells and their adaptive dynamics in type 2 diabetes

**DOI:** 10.1002/imt2.70060

**Published:** 2025-06-24

**Authors:** Xueqin Xie, Changchun Wu, Fuying Dao, Kejun Deng, Dan Yan, Jian Huang, Hao Lyu, Hao Lin

**Affiliations:** ^1^ Department of Clinical Laboratory, Sichuan Clinical Research Center for Cancer, Sichuan Cancer Hospital & Institute, Sichuan Cancer Center, School of Life Science and Technology University of Electronic Science and Technology of China Chengdu China; ^2^ School of Biological Sciences Nanyang Technological University Singapore Singapore; ^3^ Beijing Friendship Hospital Capital Medical University Beijing China

## Abstract

scRiskCell is an interpretable intelligent computational framework that leverages nearly 500,000 islet cell expression profiles from 106 donors across different continuous disease states. By calculating the intrinsic relationship between donor disease states and cell expression profiles, it assigns a pseudo‐cell state index to each cell. Sorting the pseudo‐indexes of cells enables the identification of risk cells truly disrupted by the disease. Importantly, scRiskCell reveals the dynamic aggregation pattern of risk cells during disease progression, providing mechanistic insights for early disease prediction and clinical dynamic monitoring of disease progression.

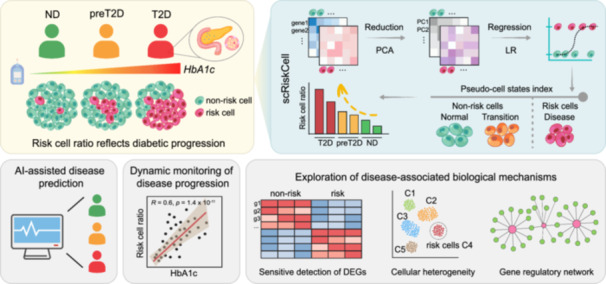

To the Editor,

Type 2 diabetes (T2D) is a chronic metabolic disorder characterized by impaired insulin secretion [1, 2]. Patients often transition through a pre‐diabetic (preT2D) phase marked by elevated blood glucose levels below diabetic thresholds [3, 4] and reduced glucose‐stimulated insulin secretion from beta cells [5, 6]. Without appropriate intervention, individuals with preT2D are at high risk of progressing to T2D. While molecular studies on diabetes exist [7, 8], the specific mechanisms driving preT2D progression remain poorly understood.

The pancreas maintains its function through a complex, heterogeneous cellular landscape. Recent studies reveal substantial functional and molecular diversity among pancreatic cell types, with beta cells further subclassified by secretory activity, identity, and maturation [9, 10]. Single‐cell RNA sequencing (scRNA‐seq) has mapped the cell‐type‐specific molecular phenotypes in islet cells between nondiabetic (ND) and diabetic individuals [11], but low sensitivity in detecting rare subpopulations, particularly epsilon cells, hinders in‐depth analysis of their functional transcriptomes. Significant intra‐ and inter‐individual heterogeneity in T2D beta cells has been observed [12, 13], suggesting coexistence of normal and dysfunctional cells. However, small sample sizes and non‐disease‐related confounders obscure subtle disease‐state distinctions. Precise characterization of rare islet cell subpopulations and their risk factors is critical for unraveling diabetic progression mechanisms.

To address this, we developed scRiskCell, a machine learning‐based computational framework to identify cell‐type‐specific risk cells in pancreatic islets, including rare epsilon cells, thereby uncovering the progression mechanisms of diabetes and the impact of these rare islet cell types. Through rigorous in‐sample and cross‐sample evaluations, we demonstrated that scRiskCell achieves robust detection of genuine disease‐perturbed risk cells across diverse cohorts, even in low‐abundance cell populations, and effectively disentangles pathological variations from technical or donor‐specific confounders. This framework not only quantifies dynamic shifts in risk cell proportions along the ND‐preT2D‐T2D continuum but also pinpoints key transcriptional programs driving beta cell dysfunction, enabling more sensitive detection of gene expression changes than donor‐based labeling and revealing alterations in gene regulatory networks (GRN) perturbed during disease progression.

## RESULTS AND DISCUSSION

### Meta‐analysis maps human islet cell diversity

**FIGURE 1:**
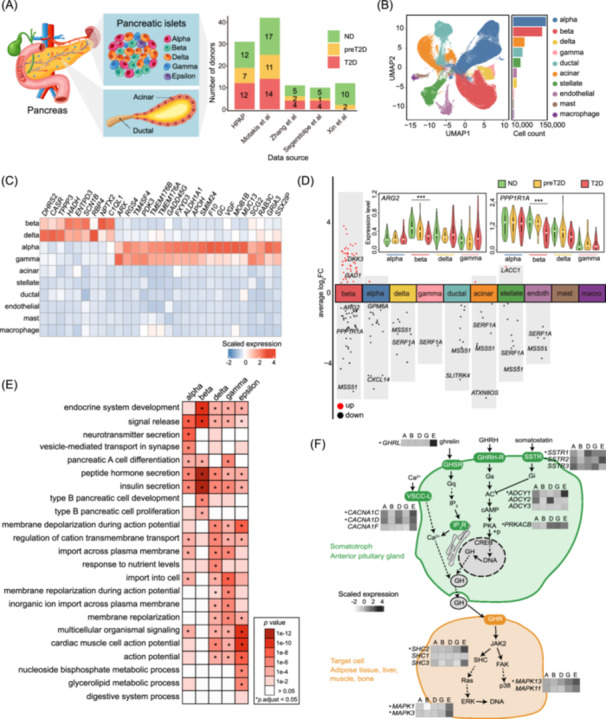
Meta‐analysis of human islets. (A) Integration of human islet scRNA‐seq for meta‐analysis. ND, nondiabetic; preT2D, pre‐diabetic; T2D, type 2 diabetes. (B) Unsupervised clustering from 495,945 pancreatic islet cells, with cell annotations visualized on UMAP coordinates. The number of cells for each cell type is displayed as a bar plot in the right panel. (C) Dual‐cell enriched markers for beta‐delta and alpha‐gamma cells. Values represent average expression after mean‐centering and scaling (*z*‐score) across cell types. Negative values indicate expression below the gene's average across cell types. (D) Scatter plots of average log_2_ fold‐change expression (*y*‐axis) detected between T2D and ND from 10 cell types (*x*‐axis) at pseudobulk level. endoth: endothelial; macro: macrophage. Genes with an adjusted *p*‐value < 0.05 were considered statistically differentially expressed. Adjusted *p*‐values were derived from pseudobulk differential expression analysis using DESeq2 with Benjamini–Hochberg (BH) correction. (Top) Violin plots highlight examples of differentially expressed genes between T2D and ND in beta cells. ***p.adj < 0.001. (E) Gene set enrichment analysis of cell‐type‐specific genes in endocrine cells. (F) Epsilon‐cell‐specific expression in growth hormone synthesis, secretion, and action pathway. Genes marked with * represent epsilon cell‐specific markers. Relative gene expression is shown in pseudo color. Values represent *z*‐score normalized average expression across cell types (mean‐centered and scaled per gene). A, alpha cell; AC, adenylate cyclase; B, beta cells; cAMP, cyclic adenosine monophosphate; CREB, cAMP‐response element binding protein; D, delta cells; E, epsilon cells; ERK, extracellular signal‐regulated kinase; FAK, focal adhesion kinase; G, gamma cell; GH, growth hormone; GHSR, GH secretagogue receptor; Gq, G protein alpha q subunit; GHRH, GH‐releasing hormone; GHRH‐R, GHRH receptor; GHR, GH receptor; Gi, G protein alpha inhibitory subunit; Gs, G protein alpha stimulatory subunit; IP_3_, inositol triphosphate; IP_3_R, IP_3_ receptor; JAK2, Janus kinase 2; PKA, protein kinase A; p38, p38 mitogen‐activated protein kinase; Ras, GTPase HRas; SSTR, somatostatin receptor; SHC, SHC‐transforming protein; VSCC‐L, voltage sensitive calcium channel l‐type.

To better characterize the intrinsic heterogeneity of islet cells, we integrated scRNA‐seq data from 495,945 pancreatic cells across 106 donors (49 ND, 23 preT2D, and 34 T2D) [14, 15] (Figure 1A, Tables S1–S7). Rigorous quality control, including doublet removal (Figure S1A–E), and joint embedding analysis identified 43 clusters (Figure S1F), independent of external factors such as study origin and donor identity (Figure S1G–I). Manual re‐annotation (Figures S1J, S2A) revealed 10 distinct cell types (Figure 1B), with consistent distribution across different datasets (Figure S2B). Marker genes enrichment confirmed annotation accuracy, and hierarchical clustering showed clear segregation of endocrine cells from other cell types (Figure S2C,D). Notably, several genes traditionally linked to one cell type (e.g., *HADH* in beta) were also enriched in others (e.g., delta), indicating shared gene expression signatures across cell types (Figure 1C, Figure S2E,F). These dual‐cell expression patterns were consistent across different disease states (Figure S3), suggesting disease‐independent regulatory mechanisms.

### Donor label‐informed analysis of islet remodeling

To investigate dynamic islet cell shifts during T2D development, we analyzed cell compositions across ND, preT2D, and T2D donors. Immune cell proportions increased progressively with disease (Figure S4A), while beta cell numbers significantly declined in T2D but remained unchanged in preT2D [16] (Figure S4B). Other cell types remained stable across diabetic progression (Figure S4B). We then performed differential expression analyses across disease groups based on the original donor labels. The most striking changes were observed in beta cells between T2D and ND, with 82 beta‐specific genes identified (Figure 1D, Figure S4C,D, Table S8). Notably, one of downregulated genes in T2D beta cells, *ARG2*, is also significantly suppressed in diabetic bulk transcriptomic data (Figure 1D). Moreover, the key gene pairs influenced by *ARG2* have been validated to accurately distinguish between ND and T2D [17]. Another prominent gene, *PPP1R1A*, plays a crucial role in beta cell function and glucose homeostasis, its downregulation may lead to reduced insulin secretion and lower insulin levels [18].

### Epsilon cells exhibit specific growth hormone and metabolic signatures

Through re‐clustering of *GHRL*+ (ghrelin‐positive) cell clusters (Figure S1J: clusters 25 and 31), we identified 91 epsilon cells characterized by high *GHRL* expression (Figure S5A–D). These rare epsilon cells demonstrated 382 enriched genes, clustered into 6 functional groups via *k*‐means analysis (Figure S5E, Table S9). Cluster 3 highlighted pathways associated with steroid hormone synthesis, amino/fatty acid metabolism, and secretion, suggesting epsilon cells may regulate systemic energy homeostasis. To gain a deeper understanding of the distinct roles from epsilon cells, we next identified cell‐type‐specific genes across various endocrine cells. Endocrine cell‐specific genes were enriched in terms related to endocrine system development, peptide hormone secretion, and signal release (Figure 1E). Notably, epsilon cells are uniquely enriched for growth hormone‐related genes, including *GHRL*, calcium channels (*CACNA1C/D*), and somatostatin receptors (*SSTR1/2*), critical for growth hormone secretion and signal integration (Figure 1F).

### scRiskCell reveals cell‐type‐specific risk profiles

**FIGURE 2:**
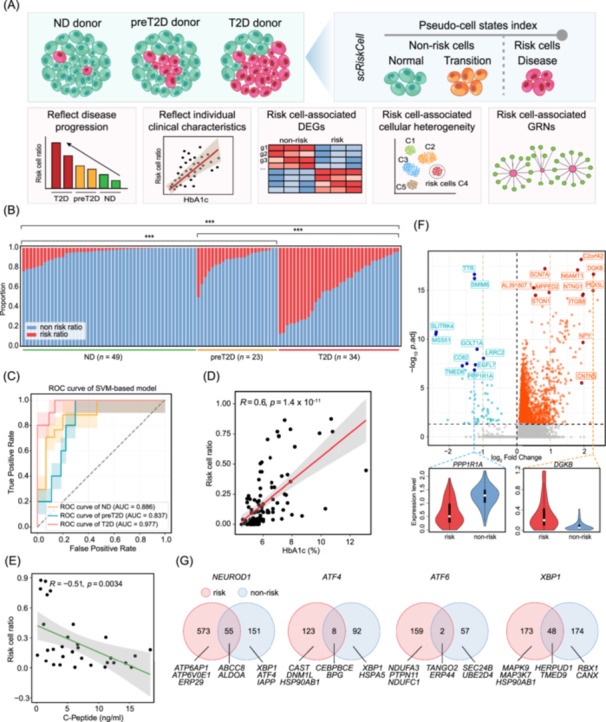
scRiskCell performance and application. (A) Schematic of scRiskCell. DEGs, differentially expressed genes; GRNs, gene regulatory networks. (B) Bar graphs demonstrating the percentage of beta risk and non‐risk cells in each donor. ND *n* = 49; preT2D *n* = 23; T2D *n* = 34. Two‐sided Wilcoxon rank‐sum test. The *p‐*values were adjusted for multiple comparisons using the Benjamini‐Hochberg (BH) method. Significance levels: ***p.adj < 0.001. (C) ROC curve of support vector machine (SVM)‐based model for disease states prediction (ND, preT2D, and T2D) using beta risk cell proportions. Shaded areas represent 95% confidence intervals estimated from 2000 bootstrap replicates. Pearson correlation between beta risk cell ratio and HbA1c (D, *n* = 105), and C‐peptide (E, *n* = 31). Pearson correlation was assessed using a two‐sided *t*‐test for the correlation coefficient. The bands around the linear regression line represent the range of 95% confidence interval of the risk cell ratio. (F) Differentially expressed genes (DEGs) at the pseudobulk level between risk and non‐risk of beta cells. Genes with an adjusted *p*‐value < 0.05 were considered statistically differentially expressed. Adjusted *p*‐values were derived from pseudobulk differential expression analysis using DESeq2 with BH correction. (Bottom) Violin plots highlight examples of DEGs with different dysregulation directions between risk and non‐risk beta cells. (G) Venn diagrams of unique/shared genes targeted by *NEUROD1, ATF4, ATF6*, and *XBP1* in risk and non‐risk beta cells. Example of genes in each category is listed at the bottom of each graph.

Studies have shown that beta cells exhibit disease‐associated cellular heterogeneity [19, 20]. To identify cell‐type‐specific risk cells truly perturbed by disease, we developed scRiskCell, a Python toolkit for single‐cell transcriptomic analysis (Figure 2A).

We firstly applied scRiskCell to beta cells and observed a monotonic increase in their disease indexes within T2D progression (Figure S6A,B). We defined beta risk cells with a disease index >0.89 and calculated their risk cell proportion per individual (Figure S6C–F). The beta risk cell proportion increased with disease progression and differed significantly among various clinical groups (Figure 2B). Using these proportions to predict disease states in 106 individuals, we found that <10% indicated ND, 10%–20% preT2D, and >20% T2D, with area under the curves (AUCs) of 0.936 (T2D vs. ND), 0.831 (T2D vs. preT2D), and 0.757 (preT2D vs. ND) (Figure 2B, Figure S6C). The machine learning‐based models using only the beta risk cell proportion further confirmed its predictive power (AUCs ≥ 0.837) (Figure 2C, Figure S6G).

To validate beta risk cells, we used 4 external datasets with varying cell counts, donor numbers, and disease states (Figure S7, Tables S10–13). In data set 1, which included preT2D donors, the beta risk cell proportion increased with disease progression (Figure S8A–D), achieving AUCs ≥ 0.914 for disease state classification (Figure S8E). For datasets without preT2D donors, the model still maintained high performance (Figure S8F–T). These findings demonstrate that the beta risk cell proportion identified by scRiskCell robustly distinguishes disease states, regardless of sample variability, and holds strong potential as a single‐feature marker for diabetic progression.

We further extended scRiskCell to other islet cells. Alpha cells showed limited ability to distinguish preT2D from T2D, though they performed well in separating ND and T2D (Figure S9A–E). Similar patterns were observed in delta and gamma cells (Figure S9F–O). Notably, epsilon risk cells showed better discrimination for preT2D (Figure S9P–T). These results may suggest a progression pattern of T2D: beta cells are early indicators in preT2D, while other islet cells become involved as the disease advances, reflecting adaptive changes in islet function.

### Risk beta cell proportion reflects key clinical parameters

To assess clinical relevance, we examined associations between beta risk cell proportion and clinical traits. Risk cell proportion was lower in younger individuals and showed an increasing trend with age, although it did not reach statistical significance (Figure S10A,B). No significant differences were observed between males and females (Figure S10C). Obese individuals (body mass index, BMI ≥ 30) had a higher beta risk cell ratio, with a significant positive correlation between BMI and risk cell proportion (Figure S10D,E). Hemoglobin A1c (HbA1c) levels positively correlated with risk cells (*R* = 0.6, *p* = 1.4 × 10^−11^), while C‐peptide showed a negative correlation (Figure 2D,E), consistent with T2D progression and beta cell dysfunction. Grouping individuals by average beta risk cell proportion revealed significant differences in BMI, HbA1c, and C‐peptide (Figure S11A). However, further stratification within disease states showed no clear subgroup differences, suggesting that additional features are needed for subtype classification (Figure S11B–D).

### Risk beta cells reveal T2D‐associated molecular signatures and regulatory networks

Transcriptomic comparison of risk versus non‐risk cells enables high‐sensitivity detection of cell‐type‐specific gene expression changes associated with T2D, especially in beta cells, where 2,734 differentially expressed genes (DEGs) were identified (Figure 2F, Figures S12A, S13A), far exceeding than those detected using donor labels (Figure S12B: 99 DEGs, with 55 overlapping DEGs). These DEGs include diabetes‐ and beta cell function‐related genes such as *HNF1A*, *DGKB*, and *IRS1* (Table S14). Similar analyses in alpha (81 DEGs), delta (1,078 DEGs), and gamma cells (4 DEGs) also uncovered risk‐specific signatures (Figure S12A–C).

To explore upstream regulation, we used pySCENIC to identify cell‐type‐specific regulons. Beta cells were enriched for *MAFA*, *NKX6‐1*, and *PDX1* (Figure S13B). A total of 103 transcription factors (TFs) were found to be differentially expressed in risk beta cells, including *FOXA3* and *HNF1A* (Figure S13C, Tables S15–16). GRN reconstruction showed that risk beta cells are dominated by endoplasmic reticulum (ER) stress‐ and apoptosis‐related TF hubs like *ATF4*, *ATF6*, and *XBP1*, which drive target genes involved in ER stress, oxidative phosphorylation, autophagy, and apoptosis (Figure 2G, Figure S13D). These findings suggest that beta risk cells undergo transcriptional reprogramming involving ER stress, metabolic dysregulation, and impaired insulin function, highlighting their role in diabetes progression.

### The dynamic aggregation pattern of risk beta subtype during diabetes progression

We identified dynamic aggregation patterns of beta risk cell subtypes during diabetes progression. While global clustering showed an overall increase in risk cells, it lacked resolution to detect subpopulation‐specific aggregation (Figure S14A). Louvain clustering of beta cells revealed 10 subclusters present across ND, preT2D, and T2D stages (Figure S14B). Risk cells began to aggregate in clusters 1 and 8 during preT2D, shifting to clusters 3, 5, 6, and 7 in T2D (Figure S14C). Clusters 5–8 showed higher proportions of T2D‐derived cells and risk cells, with cluster 5 showing the highest proportion of risk cells in T2D (62%) (Figure S14D–F).

Gene expression profiles indicated functional heterogeneity. Clusters active in preT2D (clusters 1 and 8) were associated with glucose metabolism and protein secretion, suggesting early adaptive responses (Figures S14G–H, S15). In contrast, T2D‐associated clusters (clusters 3, 5, 6, 7) showed enrichment in genes linked to ribosomal function, protein folding, autophagy, and ER stress, indicating progressive dysfunction in fundamental cellular processes (Figures S14G–H, S15). Surface marker analysis identified potential therapeutic targets (Figure S14I, Table S17). Cluster‐specific genes included *LAPTM4A/B* (cluster 1), *CASR*, *RNF145*, and *SLC2A13* (clusters 3/7), *TMEM176A/B* and *S100A10* (cluster 8), *ABCC8* and *ADIPOR1* (cluster 5), and *APP* and *ATP6AP2* (cluster 6). These findings provide a guide for annotating risk beta cell subgroups at various pathological stages, and offer insights for exploring distinct targets at different disease stages for early intervention.

## CONCLUSION

Taken together, we developed scRiskCell, an interpretable machine learning framework that leverages islet transcriptomic profiles to identify disease‐perturbed risk cells. Clinically, our study shows that risk beta cells are effective indicators for tracking diabetes progression; their proportion increases alongside worsening glycemic control and declining beta cell function. Importantly, the predictive performance of scRiskCell generalizes well across different sequencing platforms and cell types, including rare populations such as epsilon cells, underscoring its translational potential for early detection and disease staging.

## METHODS

This study presents scRiskCell, a framework designed to identify disease‐associated risk cells by ranking single cells within each cell type according to a transcriptomics‐derived disease index. Risk cells are defined using quantile or sliding window methods, and their proportions per donor serve as predictive markers for disease staging. Full technical details are provided in the Supporting Information.

## AUTHOR CONTRIBUTIONS

**Xueqin Xie**: Methodology; visualization; writing—original draft. **Changchun Wu**: Software; data curation. **Fuying Dao**: Software. **Kejun Deng**: Validation. **Dan Yan**: Validation. **Jian Huang**: Writing—review and editing. **Hao Lyu**: Conceptualization; methodology; funding acquisition; writing—review and editing. **Hao Lin**: Conceptualization; methodology; funding acquisition; writing—review and editing.

## CONFLICT OF INTEREST STATEMENT

The authors declare no conflict of interest.

## FUNDING INFORMATION

Major Project of the Fundamental Research Funds for the Central Universities, Grant/Award Number: ZYGX2024Z011; Sichuan Province Science and Technology Support Program, Grant/Award Number: 2025ZNSFSC1465; China Postdoctoral Science Foundation, Grant/Award Numbers: 2023TQ0047, GZC20230380; National Natural Science Foundation of China, Grant/Award Numbers: 62402089, 82130112, U24A20789.

## ETHICS STATEMENT

This study utilized publicly available human single‐cell databases. No direct recruitment or intervention with human subjects was involved in this study.

## Supporting information


**Figure S1:** Single‐cell RNA sequencing (scRNA‐seq) quality control and cell type annotations.
**Figure S2:** Pancreatic islet cell‐type‐specific gene expression.
**Figure S3:** Dual‐cell enriched markers for different cell types across disease status.
**Figure S4:** Cell‐type‐specific and pan‐cell differentially expressed genes (DEGs) across different clinical comparisons.
**Figure S5:** Identification of epsilon cell clusters and epsilon‐specific genes.
**Figure S6:** Sensitivity analysis of threshold selection for scRiskCell.
**Figure S7:** Data preprocessing in four validation datasets for scRiskCell.
**Figure S8:** scRiskCell identifies type 2 diabetes (T2D)‐associated beta risk cells in validation datasets.
**Figure S9:** scRiskCell identifies cell‐type‐specific risk cells associated with type 2 diabetes (T2D).
**Figure S10:** Risk beta cell proportion mirrors clinical traits.
**Figure S11:** Comparison of clinical features within disease and disease subgroups.
**Figure S12:** Molecular specific changes associated with risk cells.
**Figure S13:** Gene regulatory networks changes associated with risk cells.
**Figure S14:** Risk cell aggregation patterns of beta cell subsets in diabetes progression.
**Figure S15:** Enrichment analysis of beta subpopulation markers.

**Table S1.** Summary of human islet data collection.
**Table S2.** Donor characteristics of islet samples in public HPAP cohort.
**Table S3.** Donor characteristics of islet samples in public cohort GSE221156.
**Table S4.** Donor characteristics of islet samples in public cohort GSE195986.
**Table S5.** Donor characteristics of islet samples in public cohort E‐MATB‐5061.
**Table S6.** Donor characteristics of islet samples in public cohort GSE114297.
**Table S7.** Characteristics of donors in non‐diabetic, pre‐diabetes, and type 2 diabetes groups.
**Table S8.** Differentially expressed genes **(**DEGs) in type 2 diabetes (T2D) vs non‐diabetic (ND) groups for beta cells.
**Table S9.** Epsilon specific genes.
**Table S10.** Donor characteristics of islet samples in public cohort GSE200044.
**Table S11.** Donor characteristics of islet samples in public cohort GSE81608.
**Table S12.** Donor characteristics of islet samples in public cohort GSE153855.
**Table S13.** Donor characteristics of islet samples in public cohort GSE101207.
**Table S14.** Differentially expressed genes **(**DEGs) in risk vs non‐risk groups for beta cells.
**Table S15.** Human transcription factor (TF) list.
**Table S16.** Differentially expressed transcription factor (TF) genes.
**Table S17.** Cell surface marker list.

## Data Availability

The data and scripts used are saved in GitHub https://github.com/xiexq007/scRiskCell. The scRiskCell toolkit, along with its detailed usage instructions, is available at http://lin-group.cn/server/scRiskCell. Supplementary materials (text, methods, figures, tables, graphical abstract, slides, videos, Chinese translated version, and update materials) may be found in the online DOI or iMeta Science http://www.imeta.science/.
