# Supplementary material for: scRiskCell: A single‐cell framework for quantifying islet risk cells and their adaptive dynamics in type 2 diabetes
Source: Imeta. 2025 Jun 24;4(4):e70060. doi: 10.1002/imt2.70060 (PMC12371254; doi:10.1002/imt2.70060)
Supplement: Supplementary file 1 — Figure S1: Single‐cell RNA sequencing (scRNA‐seq) quality control and cell type annotations. Figure S2: Pancreatic islet cell‐type‐specific gene expression. Figure S3: Dual‐cell enriched markers for different cell types across disease status. Figure S4: Cell‐type‐specific and pan‐cell differentially expressed genes (DEGs) across different clinical comparisons. Figure S5: Identification of epsilon cell clusters and epsilon‐specific genes. Figure S6: Sensitivity analysis of threshold selection for scRiskCell. Figure S7: Data preprocessing in four validation datasets for scRiskCell. Figure S8: scRiskCell identifies type 2 diabetes (T2D)‐associated beta risk cells in validation datasets. Figure S9: scRiskCell identifies cell‐type‐specific risk cells associated with type 2 diabetes (T2D). Figure S10: Risk beta cell proportion mirrors clinical traits. Figure S11: Comparison of clinical features within disease and disease subgroups. Figure S12: Molecular specific changes associated with risk cells. Figure S13: Gene regulatory networks changes associated with risk cells. Figure S14: Risk cell aggregation patterns of beta cell subsets in diabetes progression. Figure S15: Enrichment analysis of beta subpopulation markers. [file IMT2-4-e70060-s001.docx]

**Supporting information to**

**scRiskCell: A single-cell framework for quantifying islet risk cells and their adaptive dynamics in type 2 diabetes**

**Running title:** scRiskCell identifies and tracks islet risk cells in diabetic progression

Xueqin Xie^1^, Changchun Wu^1^, Fuying Dao^2^, Kejun Deng^1^, Dan Yan^3^, Jian Huang^1, *^, Hao Lyu^1, *^, Hao Lin^1, *^

^1^ Department of Clinical Laboratory, Sichuan Clinical Research Center for Cancer, Sichuan Cancer Hospital & Institute, Sichuan Cancer Center, School of Life Science and Technology, University of Electronic Science and Technology of China, Chengdu, 610054, China

^2^ School of Biological Sciences, Nanyang Technological University, Singapore, 639798, Singapore

^3^ Beijing Friendship Hospital, Capital Medical University, Beijing, 100069, China

*Correspondence: hj@uestc.edu.cn (Jian Huang), [hao.lyu@uestc.edu.cn](mailto:hao.lyu@uestc.edu.cn) (Hao Lyu), [hlin@uestc.edu.cn](mailto:hlin@uestc.edu.cn) (Hao Lin)

**Contents**

[1. Supplementary Text 3](#_Toc198801812)

[Text S1: scRNA-seq meta-analysis reveals 10 distinct islet cell clusters 3](#_Toc198801813)

[Text S2: Donor label-dependent cell-type-specific changes associated with diabetes progression 4](#_Toc198801814)

[Text S3: Genes underlying growth hormone synthesis, secretion and metabolic function are specific to epsilon cells 4](#_Toc198801815)

[Text S4: scRiskCell identifies cell-type-specific risk cells associated with diabetic progression 6](#_Toc198801816)

[Text S5: The proportion of donor risk beta cells is significantly associated with clinical characteristics 8](#_Toc198801817)

[Text S6: Risk cell analysis enables high-sensitivity detection of cell-type-specific gene expression changes associated with diabetes 8](#_Toc198801818)

[Text S7: Identity of gene regulatory network (GRN) underlying ER stress-related transcriptional regulation in risk beta cells 10](#_Toc198801819)

[Text S8: Dynamic aggregation patterns of beta cell subtype-specific risk cells in diabetes progression 11](#_Toc198801820)

[Text S9: Conclusion and discussion 13](#_Toc198801821)

[2. Supplementary Methods 16](#_Toc198801822)

[2.1 Data curation 16](#_Toc198801823)

[2.2 Raw data processing and quality control 16](#_Toc198801824)

[2.3 scRNA-seq clustering and annotation 17](#_Toc198801825)

[2.4 Public data acquisition 17](#_Toc198801826)

[2.5 Differential gene expression analysis 17](#_Toc198801827)

[2.6 Gene set enrichment analysis 17](#_Toc198801828)

[2.7 Identification of epsilon cell subpopulations 18](#_Toc198801829)

[2.8 scRiskCell and identification of risk cells 18](#_Toc198801830)

[2.9 Beta cell transcriptomic heterogeneity analysis 19](#_Toc198801831)

[2.10 Gene regulatory network inference 19](#_Toc198801832)

[3. Supplementary Figures 21](#_Toc198801833)

[Figure S1. Single-cell RNA sequencing (scRNA-seq) quality control and cell type annotations. 21](#_Toc198801834)

[Figure S2. Pancreatic islet cell-type-specific gene expression. 22](#_Toc198801835)

[Figure S3. Dual-cell enriched markers for different cell types across disease status. 23](#_Toc198801836)

[Figure S4. Cell-type-specific and pan-cell differentially expressed genes (DEGs) across different clinical comparisons. 24](#_Toc198801837)

[Figure S5. Identification of epsilon cell clusters and epsilon-specific genes. 25](#_Toc198801838)

[Figure S6. Sensitivity analysis of threshold selection for scRiskCell. 26](#_Toc198801839)

[Figure S7. Data preprocessing in four validation datasets for scRiskCell. 27](#_Toc198801840)

[Figure S8. scRiskCell identifies type 2 diabetes (T2D)-associated beta risk cells in validation datasets. 28](#_Toc198801841)

[Figure S9. scRiskCell identifies cell-type-specific risk cells associated with type 2 diabetes (T2D). 29](#_Toc198801842)

[Figure S10. Risk beta cell proportion mirrors clinical traits. 30](#_Toc198801843)

[Figure S11. Comparison of clinical features within disease and disease subgroups. 31](#_Toc198801844)

[Figure S12. Molecular specific changes associated with risk cells. 32](#_Toc198801845)

[Figure S13. Gene regulatory networks changes associated with risk cells. 33](#_Toc198801846)

[Figure S14. Risk cell aggregation patterns of beta cell subsets in diabetes progression. 34](#_Toc198801847)

[Figure S15. Enrichment analysis of beta subpopulation markers. 36](#_Toc198801848)

[Reference 37](#_Toc198801849)

### 1. Supplementary Text

### Text S1: scRNA-seq meta-analysis reveals 10 distinct islet cell clusters

We integrated publicly available human islet scRNA-seq data, comprising 495,945 pancreatic cells from 106 donors, including 49 non-diabetic (ND), 23 pre-diabetic (preT2D), and 34 type 2 diabetes (T2D) individuals (**Figure 1A, Table S1–S7**). To ensure robust comparative analysis of pancreatic cells across different individuals, we implemented a doublets-filtering pipeline for scRNA-seq data based on bimodal expression of hormone genes, removing approximately 5% of potential doublets from each dataset (**Figure S1A–E**). After quality control, we constructed a joint embedding space consisting of 495,945 cells across 43 clusters (**Figure S1F**), irrespective of study origin, donor origin, donor gender, and disease status (**Figure S1G–I**). As the available cell type annotation was incomplete and inconsistent across datasets, we manually re-annotated the integrated embedding (**Figure S1J, S2A**). We identified ten common cell types, including alpha (*GCG*), beta (*INS*), delta (*SST*), and gamma (*PPY*) cells etc., with alpha and beta cells being the most abundant (**Figure 1B**). The distribution of these ten cell types was relatively consistent across the original datasets (**Figure S2B**). To ensure the optimal trade-off between batch correction and biological preservation at the level of cell types, we identified specific marker genes for each cluster. The integrated atlas clearly shows significant enrichment of known marker genes within corresponding annotated cell cluster (**Figure S2C**). Furthermore, hierarchical clustering of the top 100 highly variable genes from each cell cluster revealed a distinct endocrine cell cluster composed of alpha, beta, delta, and gamma cells, which were segregated from other cell types (**Figure S2D**). This underscores the distinct relationship among endocrine cells and further validates the accuracy of cell annotation.

In the integrated analysis, we discovered intriguing patterns of gene expression across different pancreatic cell types. Specifically, certain genes typically associated with beta cells or alpha cells were also significantly expressed in delta and gamma cells, respectively (**Figure 1C**). For example, the beta cell-specific gene *HADH*, known for its role in congenital hyperinsulinemia (CHI) due to its involvement in excessive insulin secretion, is predominantly expressed in beta cells but also shows high expression in delta cells. This observation aligns with findings by Lawlor et al. [1] and suggests a potential role of delta cells in CHI progression. Additionally, we identified a set of genes co-enriched in both beta and delta cells, including *CASR*, *TPPP3*, *DHRS2*, and *RBP4* etc. Similarly, the alpha cell-specific transcription factor (TF) *ARX*, when misexpressed, can endow cells with characteristics of both alpha and gamma cells [2], and is notably enriched in gamma cells. Moreover, *PDK3*, a key enzyme in glucose metabolism, along with other genes such as *GC*, *VGF*, and *F10*, shows dual enrichment in alpha and gamma cells. We also observed co-enrichment of certain genes in other cell pairings, such as delta and gamma cells, as well as mast and macrophage cells (**Figure S2E**). To further explore whether these dual-cell enriched markers are influenced by different disease states, we examined their expression across three conditions (ND, preT2D, and T2D). The results showed that the dual-cell enrichment phenomenon persists across all three conditions, indicating that this unique dual-cell expression pattern is robust and independent of disease status (**Figure S3**). Comparison of the dual-cell enriched markers with those reported by Lawlor et al. revealed several overlapping genes, such as *HADH* and *RBP4*, which are enriched in both beta and delta cells. Importantly, our study also identified a number of novel dual-cell enriched markers, observed in the beta-delta or alpha-gamma cell pairings (**Figure S2F**).

### Text S2: Donor label-dependent cell-type-specific changes associated with diabetes progression

Long-term T2D contributes to beta cell loss. Thus, to assess dynamic cellular redistributions, we analyzed the islet cell composition across ND, preT2D, and T2D donors. Within each donor, the distribution of cell types exhibited substantial heterogeneity, with a gradual increase in immune cell proportion as the disease progressed (**Figure S4A**). Compared to ND donors, T2D donors showed a significant decrease in the relative number of beta cells, whereas no such alterations were observed in preT2D donors (**Figure S4B**). Although previous reports suggest a trend of increased relative alpha-cell numbers in T2D donors [3], our analysis did not reveal significant differences in alpha cell numbers across three disease states. And the relative numbers of delta, gamma or other cells were similar across three groups.

To elucidate the molecular basis of cell-type-specific functional changes associated with prediabetes and T2D, we conducted clinical comparisons across three groups (preT2D vs ND, T2D vs ND, and T2D vs preT2D) based on the original donor disease labels, aiming to identify differentially expressed molecules at the pseudobulk level. The comparisons between T2D and ND revealed the most notable cell-type-specific changes, particularly in beta cells (**Figure S4C**). We identified 82 beta-specific genes in the T2D vs. ND group, with 30 genes markedly downregulated-such as *ARG2* and *PPP1R1A*-and 52 genes upregulated, including *SYT1* and *DKK3* (**Figure 1D**, **S4D–F**, **Table S8**). The inhibition of *ARG2* may be related to beta cell apoptosis, substantially reducing polyamine levels in insulin-secreting cells, thereby impairing insulin secretion [4]. Research indicates that the *PPP1R1A* gene plays a crucial role in beta cell function and glucose homeostasis, and its downregulation may lead to decreased insulin secretion and reduced insulin levels [5]. Additionally, we observed 8 alpha-specific genes (1 upregulated and 7 downregulated) and 5 stellate-specific genes (1 upregulated and 4 downregulated) in the ND vs. T2D group. Moreover, we found that *SERF1A* displayed significant alterations in T2D vs. ND comparison across all 8 cell types, except for two immune cell types, potentially representing a pan-cell-type changes associated with diabetes progression (**Figure S4G**).

### Text S3: Genes underlying growth hormone synthesis, secretion and metabolic function are specific to epsilon cells

During cell annotation, we identified clusters 25 and 31 as being enriched with a small number of epsilon cells, which exhibited expression profiles similar to ghrelin-positive cell (**Figure S1J**). Epsilon cells are among the least studied islet cell types due to their scarcity. To investigate the molecular characteristics of these cells, we performed secondary clustering on clusters 25 and 31 separately, with the aim of distinguishing epsilon cell subpopulations. Re-clustering of the *GHRL*+ cluster 25 revealed 11 distinct subclusters, which were independent of external factors such as data source (**Figure S5A–B**). Among these 11 new subclusters, cluster 10 was significantly enriched for *GHRL* rather than *SST* or *PPY* (**Figure S5C**), promoting us to reannotate this cluster as epsilon cells. Similarly, secondary clustering and reannotation of *GHRL*+ cluster 31 revealed a small subpopulation significantly enriched for *GHRL*, independent of external factors, which we also identified as epsilon cells (**Figure S5A–B, S5D**). As a result, we characterized a total of 91 epsilon cells, including 42 from healthy individuals, 27 from preT2D, and 22 from T2D samples.

Further analysis of the 91 epsilon cells identified 382 genes that were significantly enriched in these cells (*p*.adj < 0.01 & avglog_2_FC > 1.5). *K*-means Clustering grouped these genes into six clusters based on their expression patterns: cluster 1 (37 genes), cluster 2 (30 genes), cluster 3 (113 genes), cluster 4 (40 genes), cluster 5 (66 genes), and cluster 6 (96 genes) (**Figure S5E**, **Table S9**). The genes in these clusters displayed distinct expression preferences. For example, genes in cluster 2 exhibited co-expression patterns between epsilon and macrophage cells, reflecting diverse biological functions. Cluster 3, which contained the largest number of genes, was primarily associated with pathways related to steroid hormone, amino acid, and fatty acid metabolism and secretion (**Figure S5E**), suggesting that epsilon cells may play a key role in regulating energy and metabolic homeostasis.

To gain a deeper understanding of the distinct roles of epsilon cells, we next identified cell-type-specific genes across various endocrine and non-endocrine cell types (*p*.adj < 0.01 & avglog_2_FC > 1). Endocrine cell-specific genes were enriched in pathways related to endocrine system development, peptide hormone secretion, and signal release (**Figure 1E**). In contrast, genes associated with non-endocrine cells were enriched in terms related to digestion, protein metabolism, and immune regulation. As expected, beta-cell-specific genes were linked to insulin secretion, as well as beta cell proliferation and development (**Figure 1E**). Notably, genes involved in the synthesis, secretion, and action pathway of growth hormone were highly specific to epsilon cells, including the known epsilon cell marker *GHRL*, which encodes ghrelin, a potent appetite stimulant crucial for energy homeostasis and pancreatic glucose-stimulated insulin secretion. Other notable genes include *CACNA1C* and *CACNA1D*, which encode alpha1 subunits of calcium voltage-gated channel, *SSTR1* and *SSTR2*, which encode somatostatin receptors that inhibit insulin and glucagon secretion (**Figure 1F**). Adenylate cyclase 1 (*ADCY1*), an enzyme upstream of the second messenger cyclic adenosine monophosphate (cAMP), was significant enriched in epsilon cells, with co-expression in alpha cells. The *PRKACB* gene, encoding protein kinase, was enriched in delta, gamma, and epsilon cells, mediating cAMP signaling. *SHC2*, encoding SHC adaptor protein 2, was specific to epsilon cells. Additionally, *MAPK1* and *MAPK3*, encoding extracellular signal-regulated kinases (ERK), as well as *MAPK13*, encoding p38 MAP kinase, were significantly enriched in epsilon cells. These enzymes, activated by upstream kinase phosphorylation, serve as integration points for various biochemical signals, participating in processes such as proliferation, differentiation, transcriptional regulation, and development [6]. These findings highlight that despite their rarity, epsilon cells play essential roles in both islet regulation and broader systemic functions, integrating systemic signals and maintaining physiological homeostasis. Gaining deeper insight into the specific functions of these rare epsilon cells and their responses to systemic cues will be crucial for understanding their roles in maintaining endocrine homeostasis and regulating glucose metabolism.

### Text S4: scRiskCell identifies cell-type-specific risk cells associated with diabetic progression

Studies have shown that beta cells exhibit disease-associated cellular heterogeneity. By assigning a pseudo ‘disease index’ that reflects the degree of disease transformation to each cell, beta cells can be further categorized into three states: normal, transition, and disease [7]. Based on this, we proposed the concept of risk cells, defining cells in the disease state as true risk cells, while others are considered non-risk cells. We hypothesized that there are cell-type-specific risk cells within human islets, with their proportion progressively increasing with individual disease progression. To validate this hypothesis, we developed scRiskCell, a Python package designed to identify disease-associated risk cells in single-cell transcriptome data (**Figure 2A**).

Initially, we applied scRiskCell to identify islet beta risk cells. We observed a monotonic increase in the disease index of beta cells with the progression of T2D, consistent with previous findings [7,8] (**Figure S6A–B**). Utilizing the *Sliding Window Method* (see *Supplementary Methods*), we set a disease index threshold of 0.89 to define risk cells and calculated the proportion of beta risk cells for each individual. The sensitivity analysis across different thresholds is shown in **Figure S6C–F**. We found that the proportion of beta risk cells increased steadily with disease progression and showed significant differences in pairwise comparisons across three clinical groups (**Figure 2B**). To verify whether the proportion of beta risk cells could discriminate disease states, we used empirical proportion data to predict the disease states of 106 individuals. For example, individuals with a beta risk cell proportion below 10% tend to be ND; those between 10% and 20% are more likely to be preT2D; and those above 20% are indicative of T2D. Through direct quantitative assessment, we achieved an area under the curve (AUC) of 0.936 for distinguishing T2D from ND, 0.831 for T2D vs. preT2D, and 0.757 for preT2D vs. ND (**Figure S6C**). Moreover, using the proportion of risk cell as a single feature, we assessed the ability of support vector machine (SVM) and logistic regression (LR) to identify ND, preT2D, and T2D samples. The result confirmed the effectiveness of the risk cell proportion feature in identifying diabetes progression states (**Figure 2C,** **S6G**).

Validation of beta risk cells for disease state recognition requires multiple perspectives. To address this, we collected four independent external validation datasets. Following a basic data annotation pipeline (**Figure S7**), we obtained four datasets representing different scenarios, including cell scale (with beta cell counts ranging from tens of thousands to a few hundred), individual disease states (with or without preT2D donors), donor numbers, and basic clinical characteristics (**Table S10–13**). For the independent dataset 1, which includes preT2D donors, we observed an increase in both the disease index and the proportion of beta risk cells with disease progression (**Figure S8A–D**). Further use of the proportion of beta risk cells to identify three disease states consistently yielded AUC values above 0.914 (**Figure S8E**). Additionally, for datasets lacking preT2D donors, we also obtained excellent model performance (**Figure S8F–T**). These results suggest that the proportion of beta risk cell identified by scRiskCell can accurately distinguish the disease states of donors, unaffected by variations in beta cell numbers and donor quantities. Importantly, we can reasonably speculate that the proportion of beta risk cells, as a single feature, has broad potential for accurate monitoring and characterization the progression of diabetes.

Next, we applied scRiskCell to explore the capacity of other islet cell types, beyond beta cells, to serve as risk cells. For each cell type, the risk cell threshold was chosen based on its respective optimal conditions to ensure accurate classification. In alpha cells, the difference of disease index across three clinical groups were weaker, with no significant differences in the proportion of risk cell between preT2D and T2D individuals (**Figure S9A–E**), indicating that alpha risk cells alone cannot accurately identify preT2D individuals. However, the proportion of alpha risk cells showed a prominent performance in distinguishing healthy individuals from those with diabetes, with an AUC of 0.868 (**Figure S9D–E**). As expected, we observed similar phenomena in delta and gamma cells, where risk cells could not effectively characterize preT2D individuals (**Figure S9F–O**). To validate the disease-state recognition potential of rare epsilon cell types, we first identified epsilon risk cells using scRiskCell and applied this analysis to 43 individuals with detected epsilon cells (ND: *n* = 18, preT2D: *n* = 9, T2D: *n* = 16). The results showed that the proportion of epsilon risk cell performed better than alpha, delta, and gamma cells in identifying preT2D individuals, with an AUC of 0.636 (**Figure S9P–T**). Based on these findings, we obtained an evolutionary trajectory of risk cells during diabetes progression: beta cells, which fluctuate dramatically in the early stages of diabetes, represent the characteristic risk cells for preT2D states. As the disease worsens, other islet cell types are gradually affected, leading to the accumulation of cell-type-specific risk cells. Importantly, rare islet cell types, such as delta, gamma cells, and especially epsilon cells, play critical roles in maintaining overall islet function and may reflect adaptive mechanisms in the progression of diabetes.

### Text S5: The proportion of donor risk beta cells is significantly associated with clinical characteristics

Given the importance of risk beta cells in characterizing the progression of diabetes, we further explored the relationship between the proportion of risk cells and clinical characteristics. **Table S7** summarizes the average age, Body Mass Index (BMI), Hemoglobin A1c (HbA1c) levels, and the number of donors divided by gender. We first divided the 106 donors into three age groups: [20, 35) *n* = 20, [35, 50) *n* = 36, [50, 70) *n* = 50. We found that the proportion of risk beta cells in age group 1 was lower compared to the 2nd and 3rd groups (**Figure S10A**). As age increased, the proportion of risk beta cells also tended to rise, although the *p*-value did not meet statistical significance (**Figure S10B**). Additionally, there was no significant difference in the proportion of risk beta cells between male and female donors, indicating the diabetes progression based on the proportion of risk cells was gender independent (**Figure S10C**).

We further classified donors into obese (BMI ≥ 30) and non-obese (BMI < 30) groups. As expected, Obese individuals exhibited a significantly higher proportion of risk beta cells compared to non-obese individuals (**Figure S10D**), with a positive correlation between BMI and the proportion of risk beta cells (**Figure S10E**). A similar trend was observed with HbA1c, where we also found a positive correlation between the proportion of risk beta cells and HbA1c, with a correlation coefficient of 0.6 (**Figure 2D**). C-peptide, a marker of beta-cell secretory activity, showed a negative correlation with the proportion of risk beta cells (**Figure 2E**). In summary, our results demonstrate that the proportion of risk beta cells increases with the progression of diabetes, aligning with the loss of glycemic control and declining beta cell function.

Subsequently, we divided all individuals (*n* = 106) into high-risk and low-risk groups based on the mean proportion of risk beta cells. We found significant differences between these two groups in terms of BMI, HbA1c, and C-peptide levels: high-risk individuals exhibited higher BMI and HbA1c and lower C-peptide levels (**Figure S11A**). To explore the potential of risk cell proportion in identifying T2D subtypes, we first categorized individuals into three groups based on their disease status, then further subdivided them into high-risk and low-risk subgroups. Unfortunately, we found no significant differences between subgroups in terms of age, BMI, HbA1c, and C-peptide levels (**Figure S11B–D**). This suggests that additional information beyond the proportion of risk beta cells is necessary for effective subtype classification within disease groups.

### Text S6: Risk cell analysis enables high-sensitivity detection of cell-type-specific gene expression changes associated with diabetes

To gain deeper insights into the molecular basis of risk cells, we compared the transcriptomic profiles of risk and non-risk cells using the obtained risk cell labels at the pseudobulk level to identify cell-type-specific changes associated with corresponding risk cells. In islet beta cells, we identified 2,734 differentially expressed genes (DEGs), of which 2,679 were upregulated and 55 downregulated (*p*.adj < 0.05) (**Figure 2F**, **S12A**). Notably, the number of DEGs identified using risk cell labels (2,734 DEGs) was significantly higher than that obtained using the original donor disease label (99 DEGs), with 55 overlapping DEGs identified as key diabetes-associated molecules (**Figure S12B**). Further analysis revealed that these key genes include *PPP1R1A*, *HNF1A*, *TTR*, *DGKB*, and *GAD1* etc., which exhibit consistent dysregulation trends in both risk cells and T2D samples (**Table S8, S14**). Among them, *HNF1A* is known to regulate tissue-specific gene expression in pancreatic and liver cells, with mutations directly linked to maturity onset diabetes of the young (MODY3, MODY1) and T2D risk [9]. Moreover, *HNF1A* deficiency in T2D has been reported to drive diabetes-related beta cell heterogeneity [7]. *DGKB* encodes diacylglycerol kinase, a regulator of intracellular concentrations of the second messenger diacylglycerol, considered a T2D risk locus and associated with impaired glucose-stimulated beta cell function [10]. Intriguingly, our analysis identified key molecules distinct from donor disease labels, including markers of beta cell aging and senescence: *CDKN2A*, *CDKN2B*, *CDKN2C*, etc.; diabetes-associated genes: *KLF11*, *IRS1*, *RBP4*, *FOXA3*, *CACNA1E*, *BLK*, *KLF14*, etc.; and genes involved in glucose metabolism: *PKLR*, *SLC2A12*, *HK2* etc. (**Table S14**). *IRS1* plays a crucial role in insulin signaling, with gene mutations associated with T2D and insulin resistance susceptibility [11]. *BLK* encodes a Src family tyrosine kinase that stimulates insulin synthesis and secretion, with mutations linked to MODY and beta cell dysfunction. *PKLR* encodes pyruvate kinase, a rate-limiting enzyme in glycolysis, while *HK2* encodes hexokinase, which catalyzes the first step of glucose metabolism by phosphorylating glucose to produce glucose-6-phosphate, both play critical roles in glucose metabolism.

In alpha cells, we identified 81 DEGs (50 upregulated and 31 downregulated) that showed significant differences between risk and non-risk cells (**Figure S12A**). Specifically, 76 out of 81 unique DEGs were identified using the risk cell label, with only 5 overlapping with DEGs identified using the original donor label (**Figure S12B**). We found supporting evidence for the biological functions of these DEGs in relation to diabetes progression. Among them, *ADCYAP1* is involved in glucose homeostasis and induces insulin secretion via pancreatic beta cells and was significantly downregulated in alpha risk cells. Additionally, genes such as *LEPR*, which encodes the leptin receptor, and *PFKFB4*, which encodes 6-Phosphofructo-2-Kinase and plays a key role in glycolysis regulation, were upregulated in alpha risk cells (**Figure S12C**).

For delta and gamma cells, we also conducted differential analysis between risk and non-risk cells (**Figure S12A–C**). In delta risk cells, we identified 1,078 DEGs (1,074 upregulated and 4 downregulated), significantly exceeding the number of DEGs identified using donor labels (**Figure S12B**). Notably, *LAPTM4B* was significantly upregulated in delta risk cells (**Figure S12C**), which is consistent with the increased *LAPTM4B* expression in T2D delta cells reported by Segerstolpe et al. [12] Due to the limited number of gamma and epsilon cells, we detected only 4 DEGs in gamma risk cells risk cells (**Figure S12C**), with no specific findings in epsilon risk cell.

Additionally, we observed that certain hormone markers tended to be significantly downregulated in different types of risk cells. For instance, *INS* and *PPY* were downregulated in alpha risk cells, while *SST* was downregulated in delta risk cells (**Figure S12C**). Interestingly, in three of the four cell types (excluding gamma cells), the number of upregulated DEGs in risk cells was significantly exceeded the number of downregulated genes, which is associated with divergent dysregulation directions in biological processes (**Figure S13A**). Overall, our risk cell-based analytical strategy uncovers more cell-type-specific gene expression changes associated with diabetes progression and provides higher sensitivity for detecting key molecular markers. Despite the limited number of some rare cell types, our analysis confirms the research value of identifying key molecules related to diabetes progression based on such cells.

### Text S7: Identity of gene regulatory network (GRN) underlying ER stress-related transcriptional regulation in risk beta cells

To identify risk cell-specific TFs and GRNs, we analyzed a list of 1,892 human TF genes (**Table S15**) to examine their differential expression between risk and non-risk beta cells. A total of 103 TF genes showed significant differential expression, with 6 TF genes downregulated in risk cells (**Figure S13C, Table S16**). *FOXA3*, known as a “pioneer” factor, plays a crucial role in endoplasmic reticulum (ER) stress and maintaining lipid and glucose homoeostasis [13]. Moreover, *FOXA3*, together with *HNF1A*, participates in regulatory pathways that govern beta cell development. Among the upregulated TFs, *NR1D1* stands out as a key regulator of the circadian clock and metabolism. It modulates glucagon release in pancreatic alpha-cells, as well as regulates proliferation, glucose-stimulated insulin secretion, and the expression of key lipogenic genes in pancreatic alpha-cells through the AMPK-NAMPT-SIRT1 pathway [14].

The development and maturation of human beta cells depend on the coordinated activity of various TFs, each targeting distinct genomic regions to form a GRN that maintains the identity and function of normal beta cells. We applied pySCENIC [15] to scRNA-seq data from eight cell types in integrated ND human pancreas samples to identify cell-type-specific regulons (**Figure S13B**). We found that *POU5F1B* and *HOXA10* are pan-regulons across all eight cell types, while *NEUROD1* and *MAFB* are specific to endocrine cells, and *FOXC1* and *TFAP2A* are active in non-endocrine cells. Additionally, we identified several cell-type-specific regulons: *MAFA, NKX6-1,* and *PDX1* are enriched in beta cells; *CEBPA* and *EOMES* are specific to acinar cells; *SMAD1* and *CREM* are active in endothelial cells; *LEF1* and *EBF1* in stellate cells; and *ZNF548, BARX2*, and *FOSL2* are enriched in ductal cells.

Next, we applied pySCENIC to beta risk cells and beta non-risk cells to infer TF activity and construct GRNs with specific TFs. Consistent with the findings of Shrestha et al. [16], non-beta-specific regulons such as *JUND, XBP1*, and *NEUROD1* form interconnected TF hubs, whereas beta-specific TFs like *MAFA* and *PDX1* exert less influence (**Figure S13D**). Compared to beta non-risk cells, we observed that the GRN of beta risk cells is predominantly centered around *JUND* and *NEUROD1*. Additionally, ER stress-related specific regulons, such as *ATF4, ATF6*, and *CREB3L2*, are activated in risk cells, as evidenced by a significant increase in their target genes. The number of target genes associated with diabetes-related TFs, such as *NEUROD1, RFX6, HNF1A*, and *KLF11* also increases in risk cells (**Figure 2G**). Interestingly, the expression level of the *HNF1A* gene itself is significantly downregulated in beta risk cells (**Figure S13C**). In addition to having a broader scope of target genes, these specific TFs also show substantial differences in their target genes compared to non-risk cells. The regulons of *NEUROD1* and *ATF6* in risk cells are involved not only in ER stress and unfold protein responses (such as *ERP29*) but also in oxidative phosphorylation and cellular respiration (such as *ATP6AP1*, *NDUFA3*), which are notably differed from non-risk beta cells. Similar phenotypes were observed for the *XBP1* and *ATF4* regulons. In addition to ER stress response (e.g., *HSP90AB1*), the regulons of *ATF4* and *XBP1* in risk cells are also associated with autophagy and apoptosis pathways (e.g., *CAST, MAPK9*), which are absent in non-risk cells (**Figure 2G**). These findings characterize the overall dynamics of beta risk cells, indicating that ER stress, cellular metabolism, autophagy, and apoptosis are key features of beta risk cells. The restructuring of the GRN may be associated with reconfiguration of TF regulons necessary for an adequate ER stress response.

### Text S8: Dynamic aggregation patterns of beta cell subtype-specific risk cells in diabetes progression

In the initial global clustering results, we observed an increase in beta risk cells with disease progression; however, no specific aggregation of a particular risk cell subpopulation was detected (**Figure S14A**), possibly due to the limited resolution of global clustering. To better understand the relationship between risk cells and beta cell transcriptional heterogeneity, we performed Louvain clustering on beta cells and identified 10 distinct clusters (**Figure S14B**), each present in ND, preT2D, and T2D states. By mapping the distribution of risk beta cells across the three disease stages, we found that certain beta cell subclusters with risk cell aggregation were present in both preT2D and T2D groups. Specifically, in the preT2D state, clusters 1 and 8 were enriched with more risk cells. As the condition evolved, clusters 3, 5, 6, and 7 showed an increase in risk cell aggregation (**Figure S14C**). We quantified the cell ratios within these 10 clusters in ND vs. preT2D vs. T2D states and the non-risk vs. risk cell ratios (**Figure S14D**–**E**). We found that clusters 5, 6, 7, and 8, which had a high degree of risk cell aggregation, had the highest proportion of cells from T2D donors. Additionally, cluster 9 also contained a higher proportion of T2D cells. Clusters 1, 3, 5, 6, 7, and 8 had relatively high percentages of risk cells, mainly because these clusters had a higher number of cells from T2D donors, with clusters 5 and 7 having notable proportions of risk cells. We further analyzed the proportion of risk cells in each cluster across the three states. Consistent with the aggregation patterns, clusters 1 and 8 had the highest risk cell proportions in the preT2D state. In the T2D state, risk cells were predominantly found in clusters 5, 3, 6, and 7. Notably, cluster 5 exhibited a risk cell proportion as high as 0.62 in T2D state (**Figure S14F**).

To gain deeper insights into the molecular differences among beta cell subclusters, particularly those exhibiting risk cell aggregation, we identified genes significantly overexpressed in each beta subclusters (adjusted *p*-value < 0.05 and avglog_2_FC > 0.5). We found that cluster 5 was enriched with genes predominantly related to ribosomal function, whereas cluster 6 was enriched with genes associated with heat shock proteins and ER function (**Figure S14G**). We then performed enrichment analysis on these differentially expressed genes. Markers of cluster 1 were primarily linked to cellular glucose metabolism and oxygen response; cluster 8 appeared to represent a subgroup involved in protein/peptide transport/secretion; clusters 3 and 7 were similar, with markers related to protein modifications such as phosphorylation and ubiquitination, as well as endosomal/cytosolic transport and autophagy. Cluster 5, which exhibited the highest aggregation of risk cells in T2D state, was notably associated with ribosomal functions, including translation and ribosome assembly. Cluster 6, on the other hand, was primarily involved in protein folding, ER stress response, and unfolded protein response (**Figure S14G–H, S15**). Our findings suggested that in the preT2D stage, beta cell subgroups associated with cellular metabolism and peptide/protein secretion/transport are first affected by the disease, as proved by the early aggregation of risk cells. These changes in metabolic and secretory functions might reflect the body’s attempts to maintain glucose homeostasis and cope with metabolic stress by adjusting its metabolism and secretion activities. However, as the disease progresses, these compensatory mechanisms may be insufficient to handle the ongoing metabolic stress, leading to greater impacts on beta cell subgroups involved in more basic cellular physiological processes such as ribosomal function, protein modification, autophagy, and ER stress response during the diabetes stage. This shift in impact might be related to the heterogeneity of beta cell functions between the preT2D and T2D stages. These results emphasize that diabetes treatment strategies should be adjusted according to different progression stages. For instance, in the preT2D stage, treatment goals might focus more on improving cellular metabolism and secretory functions. In the diabetes stage, there may be a greater need to protect mechanisms that maintain cellular homeostasis, such as ameliorating or regulating beta cell ER stress.

To identify potential cellular markers and drug intervention targets, we examined endocrine-specific cell surface genes in these specific beta cell subgroups (**Figure S14I, Table S17**). We found that *LAPTM4B* and *LAPTM4A* were specifically expressed in clusters 1. Genes such as *CASR* and *SLC2A13* were enriched in clusters 3 and 7, while *PAM*, *ALCAM*, and *FNDC3A* were exclusively enriched in cluster 7. Additionally, *TMEM176A*, *TMEM176B*, and *S100A10* showed high expression levels in cluster 8. The ATP-sensitive potassium channel gene *ABCC8*, which regulates insulin secretion, and the essential hormone receptor gene *ADIPOR1*, involved in glucose and lipid metabolism, were significantly enriched in cluster 5. In cluster 6, genes such as *APP* and *ATP6AP2* were enriched. Overall, our findings provide a guide of the annotation of risk cell subgroups in in various pathological stages and offer insights for exploring distinct targets at different disease stages for early intervention.

### Text S9: Conclusion and discussion

Despite considerable efforts to unravel the molecular basis of T2D pathogenesis, understanding the evolution from preT2D to T2D remains limited. Studies have shown that, in disease states, not all cells are affected, and most cells may remain in a normal state [7,8]. This is consistent with the minimal cell-type-specific gene expression changes observed between T2D states and normal donors when comparing ND, preT2D, and T2D sample groups. Therefore, traditional analysis methods struggle to effectively detect the subtle changes that occur as a healthy individual progresses to a diseased state. Moreover, these small differences are often obscured by irrelevant confounding factors and inherent variability among individuals, further reducing the signal-to-noise ratio of disease markers. In contrast, differential expression analysis based on the “risk cell” strategy reveals more cell-type-specific changes related to diabetes progression. An intriguing hypothesis is that these risk cells mark the transition of the disease state.

To this end, we developed the scRiskCell pipeline to detect cell-type-specific risk cells associated with diabetes. We demonstrated that risk beta cells are highly effective in characterizing diabetes progression, as their proportion increases with the worsening of diabetes, aligning with deteriorating glycemic control and declining beta cell function. We also analyzed other cell types beyond beta cells, especially the rare epsilon cells. Epsilon cells, as the fifth endocrine cell type in human islets, have been hindered in functional and mechanistic studies due to their extremely low abundance. Nonetheless, studies have shown that epsilon cells can regulate beta cell function and that proliferation of epsilon cells can promote the regeneration of beta cells [17]. In our meta-analysis, the high-throughput nature of the study enabled us to identify 91 epsilon cells. Transcriptomic analysis of these epsilon cells revealed gene expression patterns, highlighting functional enrichment associated with growth hormone synthesis, secretion, and metabolism. These results clarify the key role of epsilon cells in sensing and integrating specific systemic cues to regulate islet function. Additionally, we obtained novel annotation markers for epsilon cells that could be used to further enrich and purify this cell subpopulation, facilitating more detailed molecular phenotyping and functional analyses.

It is worth noting that in this study, we observed significant associations between the proportion of risk beta cells and several clinical variables. However, we acknowledge that these variables may be influenced by additional aging- or metabolism-related factors, which could in turn affect the observed risk cell dynamics. For instance, the positive correlation between age and the proportion of risk cells may be partially modulated by age-associated biological processes such as chronic inflammation, immune senescence, or oxidative stress. Future studies integrating more comprehensive lifestyle and inflammation-related information, ideally in longitudinal cohorts, will be instrumental in clarifying the causal mechanisms underlying risk cell dynamics and their value as indicators of disease progression.

The observation that certain marker genes are co-enriched in two distinct endocrine cell types, such as β/δ, α/γ, across ND-preT2D-T2D progression, suggests the existence of a shared transcriptional or developmental program that transcends classical cell typing. For β/δ cells, their common progenitor may preserve collaborative mechanisms in metabolic or calcium signaling module that is retained in both cell types to maintain islet homeostasis or support intercellular communication [18]. Similarly, *ARX* has been shown to promote the development of cells toward an α/γ fate [18]. These disease-resistant dual-cell patterns likely represent intrinsic biological features, such as paracrine coordination and metabolic buffering, rather than stress-induced adaptations.

Previous studies have identified a metabolic stress phenotype related to ER stress in diabetic beta cells [19,20]. We observed similar stress conditions in risk beta cells. Specifically, the reconfiguration of GRN in these risk beta cells is associated with the reconfiguration of TF required for an appropriate ER stress response. Moreover, as the disease progresses, risk cells exhibit dynamic clustering patterns within beta cell subpopulations. They significantly aggregate in beta cell subgroups associated with ER stress response and unfolded protein response functions. This dynamic clustering pattern not only underscores the critical role of ER stress in the pathogenesis of diabetes but also suggests the adaptation and evolution of risk cells across different stages of the disease. More importantly, these dynamic aggregation patterns of risk cells could serve as novel biomarkers for diabetes staging and prognosis. Identifying and quantifying the extent of risk beta cell clustering within different subpopulations can provide valuable insights for disease prediction and early intervention.

Emerging technologies like spatial transcriptomics open new avenues to decipher the spatial organization and functional roles of risk cells. Pioneering studies reveal that pancreatic islets harbor pacemaker-like “hub cells” that orchestrate insulin secretion through calcium oscillation coordination [21]. These spatially clustered hubs exhibit heightened sensitivity to metabolic/inflammatory stress, triggering islet dysfunction. Building on this, we hypothesize that beta risk cells may adopt non-random spatial patterns, for instance, clustering near vasculature or at islet-exocrine interfaces. Such spatial preferences could enhance microenvironmental sensing (e.g., hypoxia detection) and cross-compartment communication. Spatial multi-omics mapping of these cells will elucidate their spatially driven mechanisms in T2D progression, informing precision therapeutic targeting. Moreover, diabetes frequently leads to diverse complications that significantly impact patients’ quality of life and clinical outcomes [22]. While this study focuses on risk-associated cellular signatures in disease progression, their potential for predicting complications warrants attention. Notably, β-cell dysfunction shows close links to retinopathy, nephropathy, and cardiovascular events. Evaluating key cellular signatures may enable early complication detection. Future studies will systematically investigate the mechanistic connections between risk-associated cellular signatures and complications, aiming to validate their clinical utility.

This study has some limitations. First, the cross-sectional design limits insights into the dynamic evolution of risk-associated cellular signatures, necessitating longitudinal cohorts to delineate their spatiotemporal relationships with disease progression. Moreover, future integration with spatial transcriptomics could provide additional mechanistic insights into the microenvironmental context of risk-associated cells. Further validation through large-scale prospective studies and multi-center collaborations will be essential to establish clinical translatability.

In summary, we have characterized cell-type-specific risk cells associated with diabetes progression. Our study provides a systematic framework for understanding the progression of complex diseases. The scRiskCell approach can be extended to other complex diseases, using the risk cell strategy to facilitate in-depth analysis of the genetic mechanisms associated with diseases.

### 2. Supplementary Methods

### 2.1 Data curation

We accessed publicly available human islet scRNA-seq data and metadata for 106 donors from the Human Pancreas Analysis Program (HPAP), the National Center for Biotechnology Information (NCBI) Gene Expression Omnibus (GEO), and ArrayExpress [7,12,23,24] (**Table S1**). These donors include 49 ND individuals, 23 with preT2D, and 34 with T2D. The samples were obtained from multiple resource centers, including the Scharp-Lacy Research Institute, the Southern California Islet Cell Resource Center, the University of Wisconsin, Prodo Laboratories Inc., and City of Hope National Medical Center. Detailed characteristics, such as age, sex, BMI, HbA1c and ethnicity, along with available clinical information for individual donors, are provided in the original publications and detailed in **Table S1–S6**. Data from potentially overlapping donors were excluded based on unique donor characteristics. The mean BMI, HbA1c, and age, along with number of donors categorized by sex within each disease group, are summarized in **Table S7**. Based on medical records and HbA1c levels, we reassigned donor labels into ND, preT2D, and T2D groups. Donors previously diagnosed with T2D or with an HbA1c ≥ 6.5 were classified as T2D; those without a prior T2D diagnosis but with 5.7 ≤ HbA1c ≤ 6.4 were classified as preT2D; and those without a previous T2D diagnosis and with an HbA1c ≤ 5.6 were classified as ND. The classification criteria were adapted from those used in the original studies, resulting in the reclassification of certain donors.

To validate the risk cell analysis method proposed in this study, we also obtained additional validation datasets from four independent cohorts [3,8,25,26] (**Table S1**). The characteristics of individual donors for these validation datasets are listed in **Table S10–S13**. We applied the same classification criteria to redefine and reassign labels for donors in these validation datasets.

### 2.2 Raw data processing and quality control

Raw reads from HPAP were processed using the CellRanger pipeline (v.7.2.0) provided by 10X Genomics Inc. This pipeline facilitated sample demultiplexing, alignment, filtering, and unique molecular identifier (UMI) counting. The human reference genome GRCh38 was employed for alignment. For the raw reads obtained from ArrayExpress (accession numbers: E-MTAB-5061) and GEO (accession numbers: GSE81608), sequence quality was initially assessed using FastQC (v.0.12.1) and MultiQC (v.1.22.2). Subsequently, Fastp (v.0.23.4) was utilized to trim adapters and remove low-quality sequences. After quality control, cells with less than 85% of bases achieving a Q30 quality score were excluded from further analysis. Specifically, this resulted in the removal of 196 low-quality cells from E-MTAB-5061 and 13 cells from GSE81608. The purity-filtered reads were then aligned to the human GRCh38 reference genome using the STAR aligner (v.2.7.11a) with default parameters. Gene read counts were quantified using HTSeq (v.2.0.2).

### 2.3 scRNA-seq clustering and annotation

The Seurat R package (v.5.0.1) was utilized for the filtering, integration, and analysis of the datasets. Initially, a separate Seurat object was created for each dataset. Cells were filtered to include only genes expressed in three or more cells, while excluding low-quality cells (cells with fewer than 200 genes or more than 8000 genes, mitochondrial percent > 25%). Gene expression data within each Seurat object was normalized by scaling the library size to 10,000 molecules per cell, followed by log transformation. To identify and remove potential doublets, hormone marker genes (*GCG, INS, SST*, and *PPY*) were used. Cells exhibiting high expression levels of two or more hormone genes were removed. Expression thresholds for each hormone gene were set independently based on the bimodal distribution of their log expression levels. Subsequently, the five datasets were further normalized, merged, and batch-corrected using the merge and *IntegrateLayers* functions from the Seurat package. The ‘HarmonyIntegration*’* method within the *IntegrateLayers* function was selected to adjust for batch effects across different experiments. Downstream clustering analysis was then performed using the harmony-corrected components. Clustering was conducted using Seurat's Louvain algorithm (resolution = 0.9), and cell types were annotated based on known cell markers [27,28] and visualized using Uniform Manifold Approximation and Projection (UMAP) of marker genes.

### 2.4 Public data acquisition

The transcription factor lists, motif enrichment database, and motif annotation files were obtained from the cisTarget resources website of the Aerts Lab (https://resources.aertslab.org/cistarget/). The list of cell surface proteins was downloaded from a previous study [29].

### 2.5 Differential gene expression analysis

In single-cell differential expression analysis, treating each cell as an independent replicate may overlook the inherent correlations between cells originating from the same sample. Squair et al. [30] have demonstrated that such analyses can result in numerous false positive associations. Therefore, in this study, we used pseudobulk data for differential expression analysis. We began by aggregating gene counts for each cell type across all cells from the same donor using Seurat *AggregateExpression* function, effectively performing pseudobulking based on donor ID. This generated a gene expression profile for per sample and cell type. Subsequently, we conducted differential expression analysis at the pseudobulk (sample) level using DESeq2 (v.1.38.3), with the “test.use” parameter from *FindMarkers* function set to “DESeq2”. This approach treats samples, rather than individual cells, as independent observations. Adjusted *p*-values were calculated by DESeq2 using the Benjamini-Hochberg method to control the false discovery rate (FDR). We considered genes with an adjusted *p*-value of < 0.05 to be differentially expressed.

### 2.6 Gene set enrichment analysis

We performed Gene Ontology (GO) enrichment analysis and Kyoto Encyclopedia of Genes and Genomes (KEGG) pathway analysis using the clusterProfiler [31] (v.4.11.1) R package. GO enrichment analyses were conducted with the *enrichGO* function, and KEGG pathway enrichment was carried out using the *enrichKEGG* function, both with default settings.

### 2.7 Identification of epsilon cell subpopulations

In the initial round of cell clustering and annotation, we used Seurat *FindMarkers* function to identify marker genes for each cell cluster. Drawing upon existing knowledge and literature, we defined ten common cell types: alpha, beta, delta, gamma, ductal, acinar, stellate, endothelial, mast, and macrophage cells. Due to the scarcity of cells in the sample, we did not observe a distinct epsilon cell cluster in the UMAP from the first round of clustering. However, we clearly detected the expression of the epsilon cell hormone gene *GHRL* in subclusters 25 and 31 (**Figure S1J**). Consequently, we focused on these subpopulations for a second round of cell clustering and annotation. We employed the Louvain algorithm (resolution = 0.9) in Seurat, and using *GHRL* hormone gene as a criterion to delineate epsilon cell subpopulations.

### 2.8 scRiskCell and identification of risk cells

We developed scRiskCell as a novel approach to identify disease-associated risk cells in diabetes. Unlike traditional methods that classify cells based on donor status, scRiskCell treats all cells from donors across various disease states equally within each cell type. It reorders individual cells (e.g., beta cells) according to their association with disease progression. This reordered index forms a trajectory that accurately reflects the progression from normal to preT2D, and to T2D. For datasets with and without preT2D donors, scRiskCell defines different analysis workflows. In cases without preT2D donors, we first isolate the same cell type from all donors and apply principal component analysis (PCA) to reduce the dimensionality of each cell’s transcriptome. In this study, we empirically selected the top 20 principal components (PCs) for further analysis, as they effectively captured the essential features of the data. In scRiskCell, users can adjust the number of PCs to suit their specific needs. Using these 20 PCs, we constructed a logistic regression based-model, treating donor status (ND/T2D) as a binary variable. For each cell, the model computes a regression value derived from a linear combination of the 20 PCs, which we refer to as the pseudo-cell state index, or disease index. We then reorder all cells from low to high based on this index. In this binary classification framework, a higher regression value indicates a higher likelihood of the cell belonging to the positive class (T2D), and conversely for the negative class (ND). Thus, cells toward the higher end of the order are considered more likely to be risk cells associated with the disease.

scRiskCell offers two methods for defining risk cells:

*Quantile Selection Method*: After sorting all cells by their disease index in ascending order, users can choose a threshold based on a specific quantile (typically above 50%). Cells with a disease index greater than this threshold are considered risk cells. For example, in this study, when we set the threshold at the 85th percentile, corresponding to a disease index threshold is 0.84.

*Sliding Window Method*: Users can define the window size, step size, and threshold conditions. Specifically:

(i) Sort cells by disease index in ascending order.

(ii) Starting from the end (highest disease index), slide the window to the left. The window size and step can be customized.

(iii) For each step, count the number of cells from T2D donors within the window. If this count is below a specified threshold, stop sliding and take the disease index at the start (left edge) of the current window as the risk cell threshold. If the count does not meet the threshold, continue sliding.

(iv) Repeat step iii until the condition is met.

(v) Cells with a disease index greater than the determined index are classified as risk cells, while others are not.

For data with preT2D donors, we first extract all cells (e.g., all beta cells) from ND and T2D donors, perform PCA, and build the logistic regression-based model as described. Next, we process all beta cells from preT2D donors using the same PCA dimensionality reduction and apply the established regression model to determine their disease index. We then aggregate all beta cells from ND, preT2D, and T2D donors, sort them by disease index in ascending order, and apply either the quantile selection or sliding window method to define risk cells.

Lastly, we calculate the proportion of risk cells in each donor to predict the progression of diabetes. Additionally, scRiskCell also integrates two common machine learning algorithms, support vector machines (SVM) and logistic regression (LR), to predict donor disease status based on the proportion of risk cells per donor. In these classifiers, the dataset is split into training and testing sets with a 70/30 ratio, and models are built using default parameters. Both algorithms are implemented using the scikit-learn (v.1.4.2) Python package. In the three-class task involving preT2D data, the SVM-based model employs a *one-vs-rest* strategy for multi-class prediction.

scRiskCell is available at http://lin-group.cn/server/scRiskCell.

### 2.9 Beta cell transcriptomic heterogeneity analysis

Beta cell subpopulations were defined using *k*-nearest neighbours (*k*NN)-based clustering, which were constructed with the Seurat *FindNeighbors* function (using the top 15 PCs). The clustering of these beta cell subpopulations was performed using Seurat *FindClusters* function. To isolate major subpopulations of beta cells that are distinct but not rare at the transcriptomic level, we set the resolution parameter to 0.3. Subsequently, the Seurat *FindMarkers* function was used to identify genes significantly enriched in specific beta subpopulations, with a threshold of an adjusted *p*-value < 0.05. Enrichment analysis was performed to explore the potential biological functions represented by these distinct enriched genes in different beta subclusters.

### 2.10 Gene regulatory network inference

We used the pySCENIC (v.0.12.1) [15,32] method to infer transcription factor (TF) activity in islet cells and to construct TF-specific gene regulatory networks. pySCENIC predicts regulons (TFs and their target genes) from scRNA-seq data and evaluate their activity, enabling visualization of the regulatory relationships governing gene expression. The necessary files for running pySCENIC were sourced from the cisTarget resources website, as detailed in public data acquisition. After quality control and feature selection in Seurat, we converted the raw count matrix from the single-cell data into a loom file using the loompy (v.3.0.7) Python package. Next, we inferred co-expressed TFs and their target genes from the input single-cell expression matrix, using a comprehensive list of 1,892 TFs. We pruned the target genes enriched with specific motifs based on cis-regulatory clues and selectively filtered out indirect targets. In this step, we used motif-gene annotation files, specifically the ranking databases hg38__refseq-r80__500bp_up_and_100bp_down_tss.mc9nr.genes_vs_motifs.rankings.feather to capture proximal regulatory elements within 500 base pairs (bp) upstream and 100 bp downstream of the transcription start site (TSS), and hg38__refseq-r80__10kb_up_and_down_tss.mc9nr.genes_vs_motifs.rankings.feather to capture distal regulatory elements within 10kb up- and downstream of the TSS. Finally, we scored the activity of all inferred regulons using the *AUCell* function. The resulting scores were binarized according to specific thresholds, converting the matrix into a binary format (0 for OFF, 1 for ON). Both the AUCell scores and the binarized regulons activity matrix were added to the Seurat object using the *CreateAssayObject* function for downstream analysis and visualization. Heatmap of the binary regulon activity was visualized using the ComplexHeatmap (v.2.13.4) R package. Gene regulatory network diagrams for risk and non-risk beta cells were generated based on the top 10% of results based on importance reported for TF/ target association from the co-expression modules using Cytoscape (v. 3.10.2).

### 3. Supplementary Figures


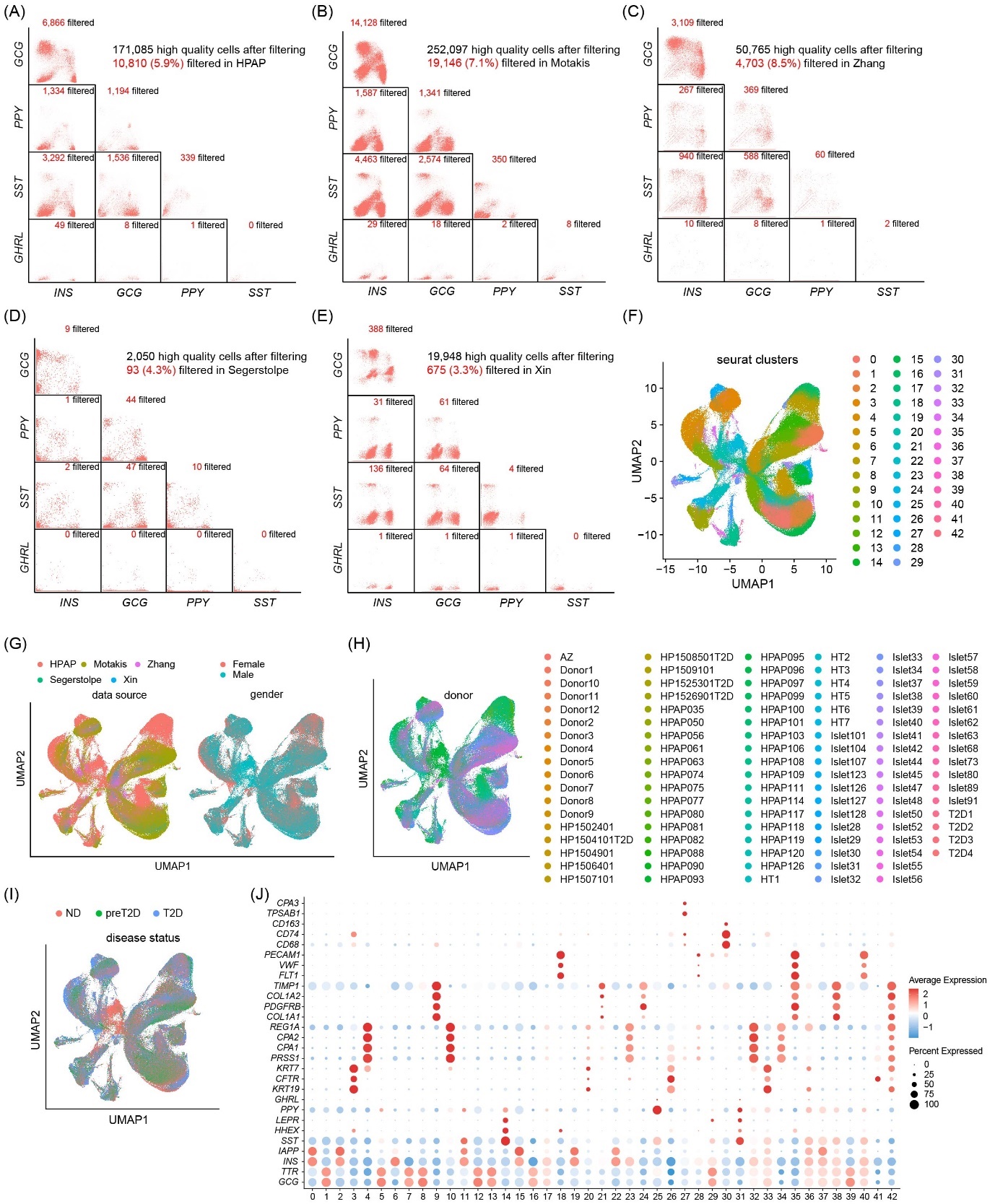


### Figure S1. Single-cell RNA sequencing (scRNA-seq) quality control and cell type annotations.

(A–E) Hormone marker-based doublets filtering strategy for different datasets. (F) Clustering of gene expression profiles from 495,945 pancreatic islet cells identifies 43 distinct clusters plotted on UMAP coordinates. (G–I) UMAP coordinates marked for each individual data source and gender (G), for donor origin (H), and for disease status (I). (J) Dot plot indicating the relative expression levels and percentage of cells in identified cell-type clusters (columns) expressing cell-type marker genes (rows).


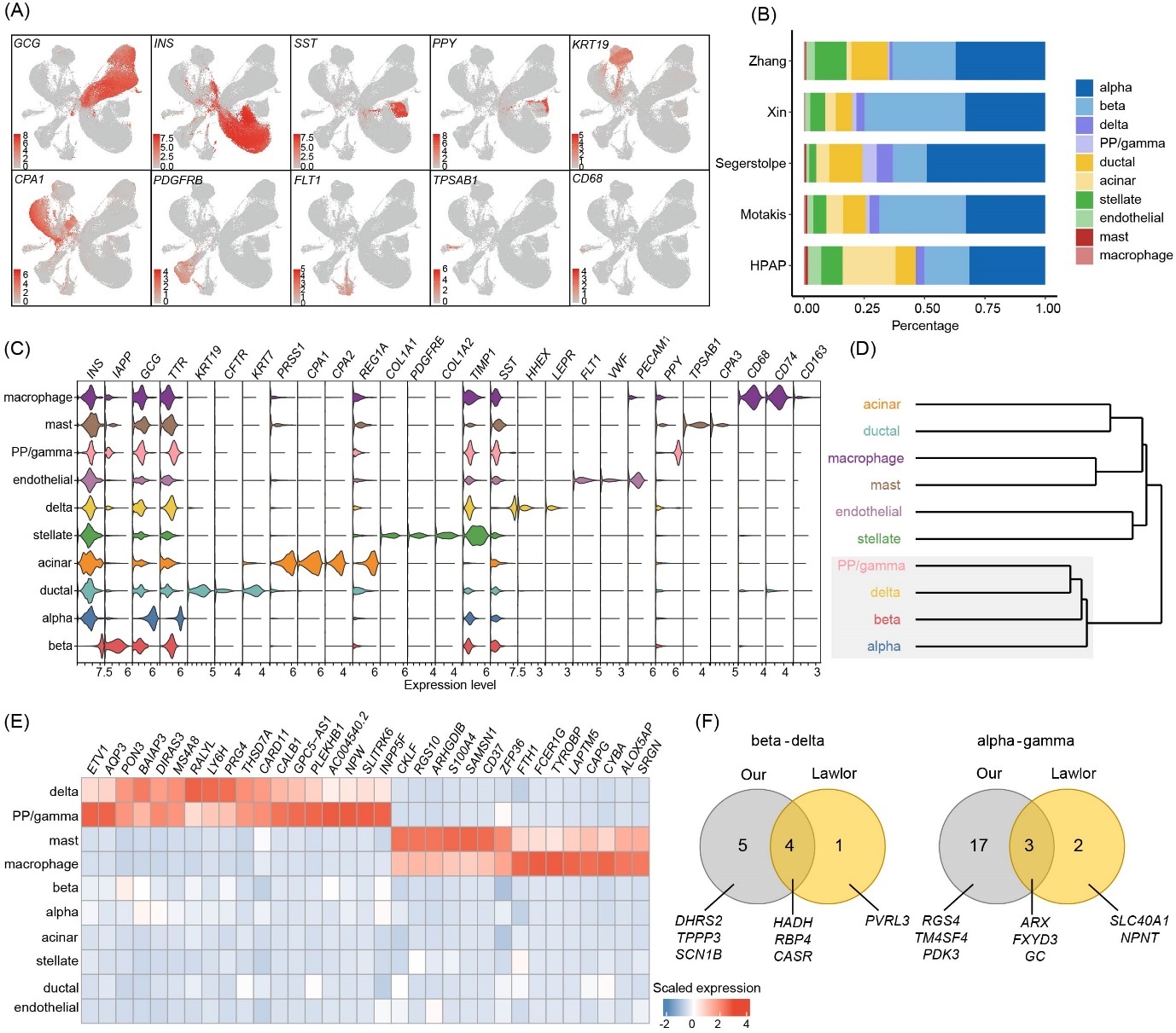


### Figure S2. Pancreatic islet cell-type-specific gene expression.

(A) RNA expression levels for selected marker genes. (B) Stacked bar graph illustrating the fraction of each cell type across different data sources. (C) Violin plots for expression of the cell-type-specific marker genes. (D) The dendrogram was computed using hierarchical clustering with average linkage on the top100 highly variable genes. (E) Dual-cell enriched markers for delta-gamma and mast-macrophage cells. Relative gene expression is shown in pseudo color. Values represent average expression after mean-centering and scaling (*z*-score) across cell types. Negative values indicate expression below the gene's average across cell types. (F) Venn diagrams showing the overlap and uniqueness of dual-cell enriched marker genes for β-δ and α-γ cell pairs identified in our study compared to those reported by Lawlor et al. Representative genes for each category are listed below the corresponding diagram.


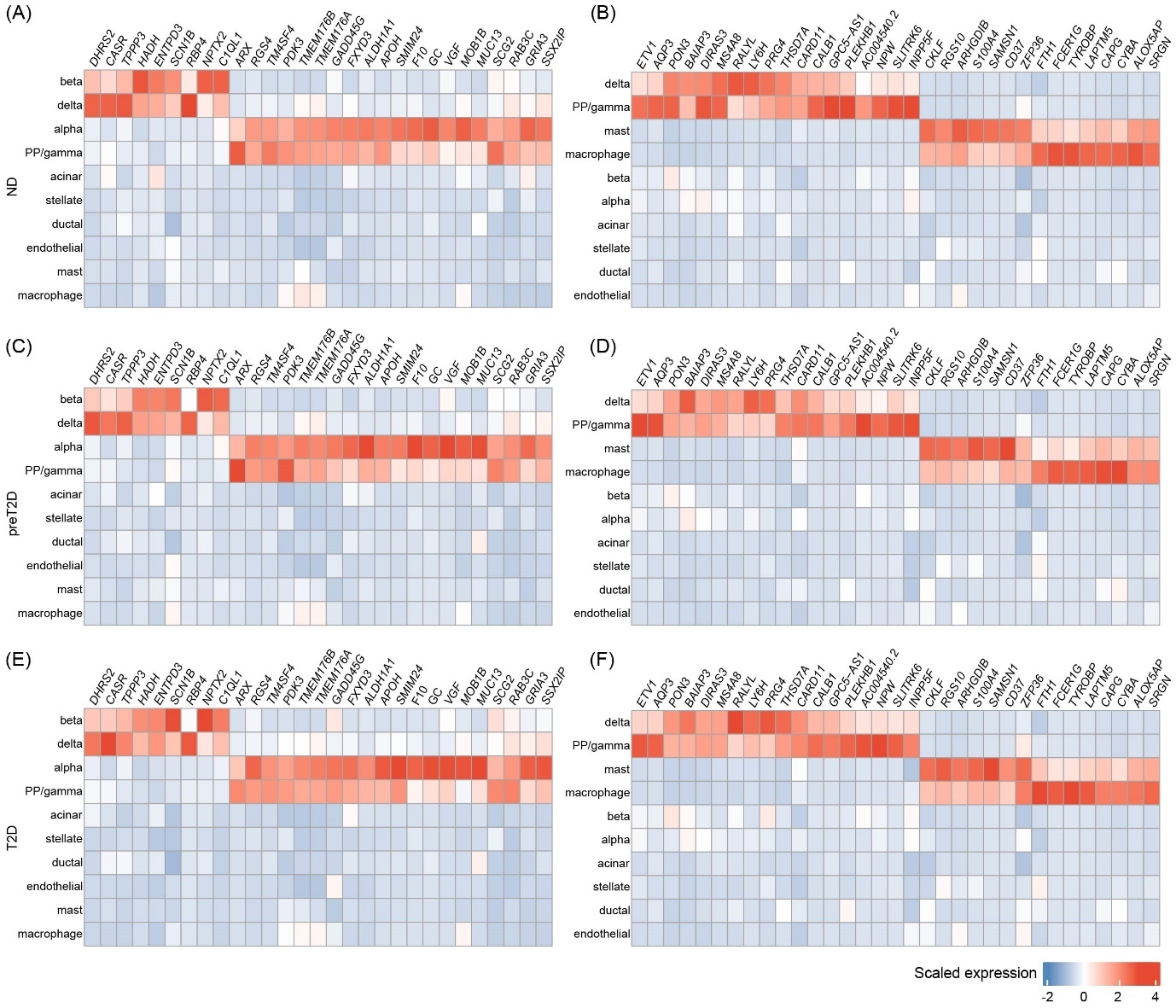


### Figure S3. Dual-cell enriched markers for different cell types across disease status.

(A–B) Dual-cell enriched markers for beta-delta and alpha-gamma cells in ND (A), for delta-gamma and mast-macrophage cells in ND (B). (C–D) Dual-cell enriched markers for beta-delta and alpha-gamma cells in preT2D (C), for delta-gamma and mast-macrophage cells in preT2D (D). (E–F) Dual-cell enriched markers for beta-delta and alpha-gamma cells in T2D (E), for delta-gamma and mast-macrophage cells in T2D (F). Relative gene expression is shown in pseudo color. Values represent average expression after mean-centering and scaling (*z*-score) across cell types. Negative values indicate expression below the gene's average across cell types.


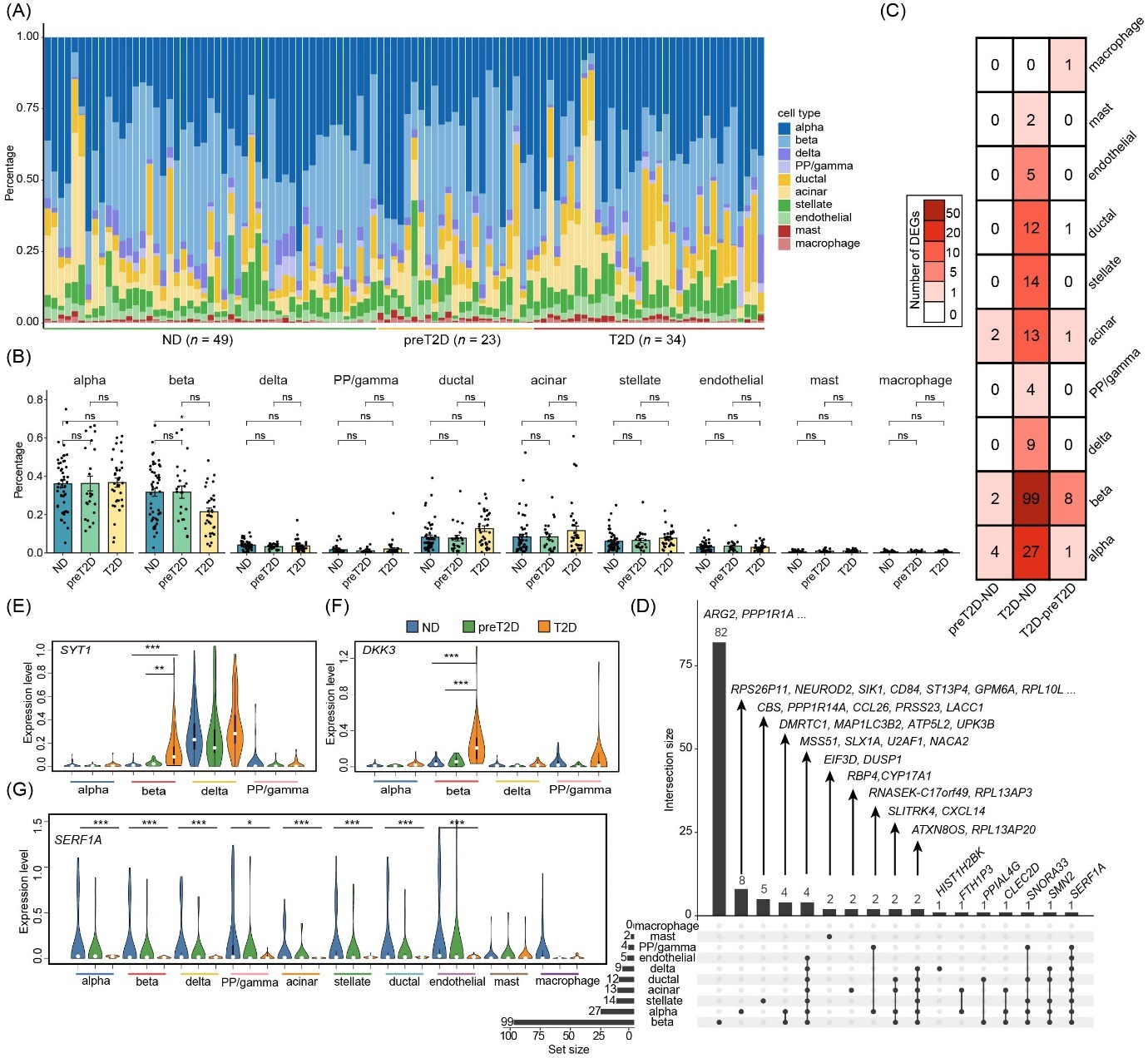


### Figure S4. Cell-type-specific and pan-cell differentially expressed genes (DEGs) across different clinical comparisons.

(A) Bar graphs demonstrating the percentage of all cell types in each donor. (B) Relative abundance of ten cell types in ND, preT2D and T2D donor islets. Data shown are the mean ± s.e.m. Dots denote data points from individual donors, with alpha, beta, delta, acinar, stellate, and ductal (ND *n* = 49, preT2D *n* = 23, T2D *n* = 34); gamma (ND *n* = 43, preT2D *n* = 21, T2D *n* = 29); endothelial (ND *n* = 49, preT2D *n* = 22, T2D *n* = 23); mast (ND *n* = 41, preT2D *n* = 22, T2D *n* = 32); and macrophage (ND *n* = 46, preT2D *n* = 21, T2D *n* = 32). Two-sided Student’s *t*-test. *P*-values were adjusted for multiple comparisons using the Benjamini-Hochberg (BH) method. Significance levels: ns *p*.adj ≥ 0.05, * *p*.adj < 0.05. (C) The number of DEGs from differential expression analysis at the pseudobulk (sample) level using DESeq2 in per clinical comparisons for each cell types. Genes with an adjusted *p*-value < 0.05 were considered statistically differentially expressed. (D) Upset plot revealed cell-type-specific DEGs and pan-cell-type DEGs in clinical comparison of T2D and ND. (E–G) Violin plots highlight examples of DEGs in different cells and comparison groups at pseudobulk level. * *p*.adj < 0.05, ** *p*.adj < 0.01, *** *p*.adj < 0.001. Adjusted *p*-values were derived from pseudobulk differential expression analysis using DESeq2 with BH correction. The long horizontal bars indicate comparisons between ND and T2D, while the short horizontal bars indicate comparisons between preT2D and T2D, across different cell types.


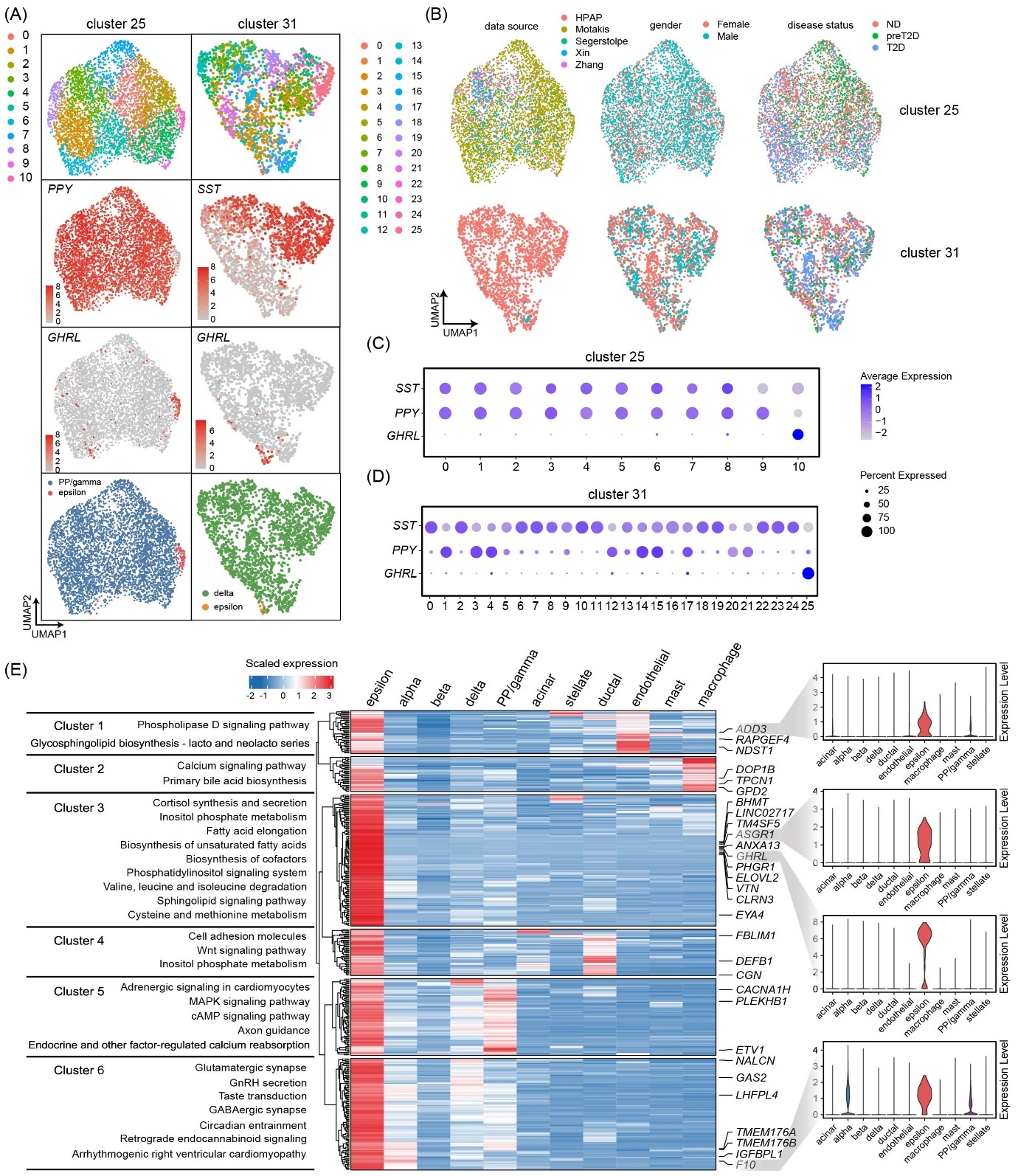


### Figure S5. Identification of epsilon cell clusters and epsilon-specific genes.

(A) Second-round clustering in clusters 25 and 31. (B) UMAP coordinates marked for data source, donor gender, and disease status in cluster 25/31 for second-round clustering. (C–D) Dot plot indicating the relative expression levels and percentage of cells in identified subclusters (columns) expressing cell-type marker genes (rows) for cluster 25 (C) and cluster 31 (D). (E) Aggregated expression level for 382 epsilon-specific genes with variable enrichment across different cells (middle). Relative gene expression is shown in pseudo color. Values represent average expression after mean-centering and scaling (*z*-score) across cell types. Negative values indicate expression below the gene's average across cell types. pathway enrichment analysis was performed for genes in each cluster and results with *p*.adj < 0.05 were considered significant (left). The violin plots illustrate representative examples of gene expression levels across various cell types (right).


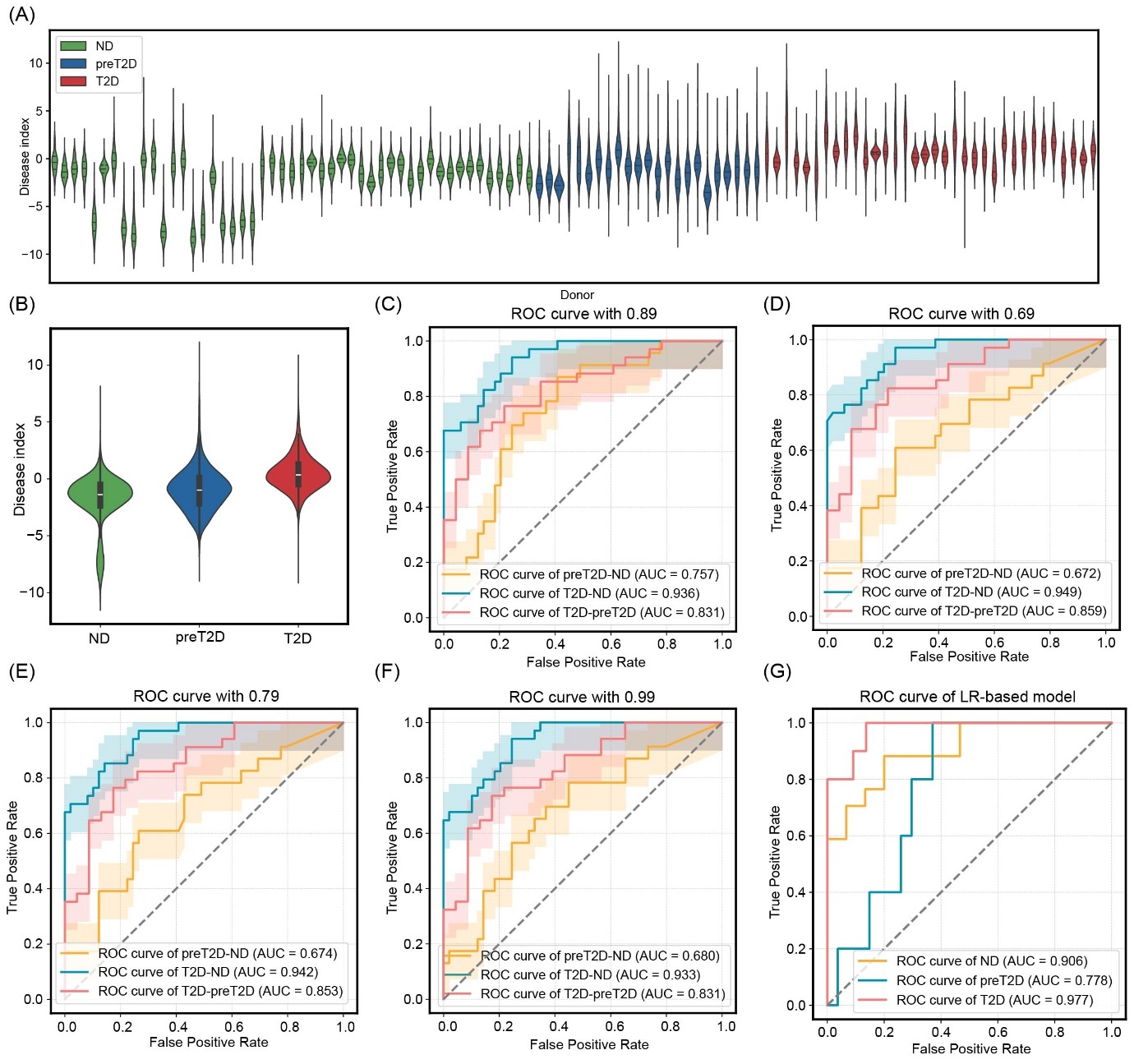


### Figure S6. Sensitivity analysis of threshold selection for scRiskCell.

(A) Violin plots showing the distribution of disease index of beta cells for each donor. Lines represent the median and quartiles. ND *n* = 49; preT2D *n* = 23; T2D *n* = 34. (B) The violin plots show disease index distribution of beta cells within three clinical groups. (C–F) Comparison of predictive performance under different threshold selections. Shaded areas represent 95% confidence intervals estimated from 2,000 bootstrap replicates. (G) ROC curve of the logistic regression (LR)-based model for disease state prediction using beta risk cell proportions. ND *n* = 49; preT2D *n* = 23; T2D *n* = 34.


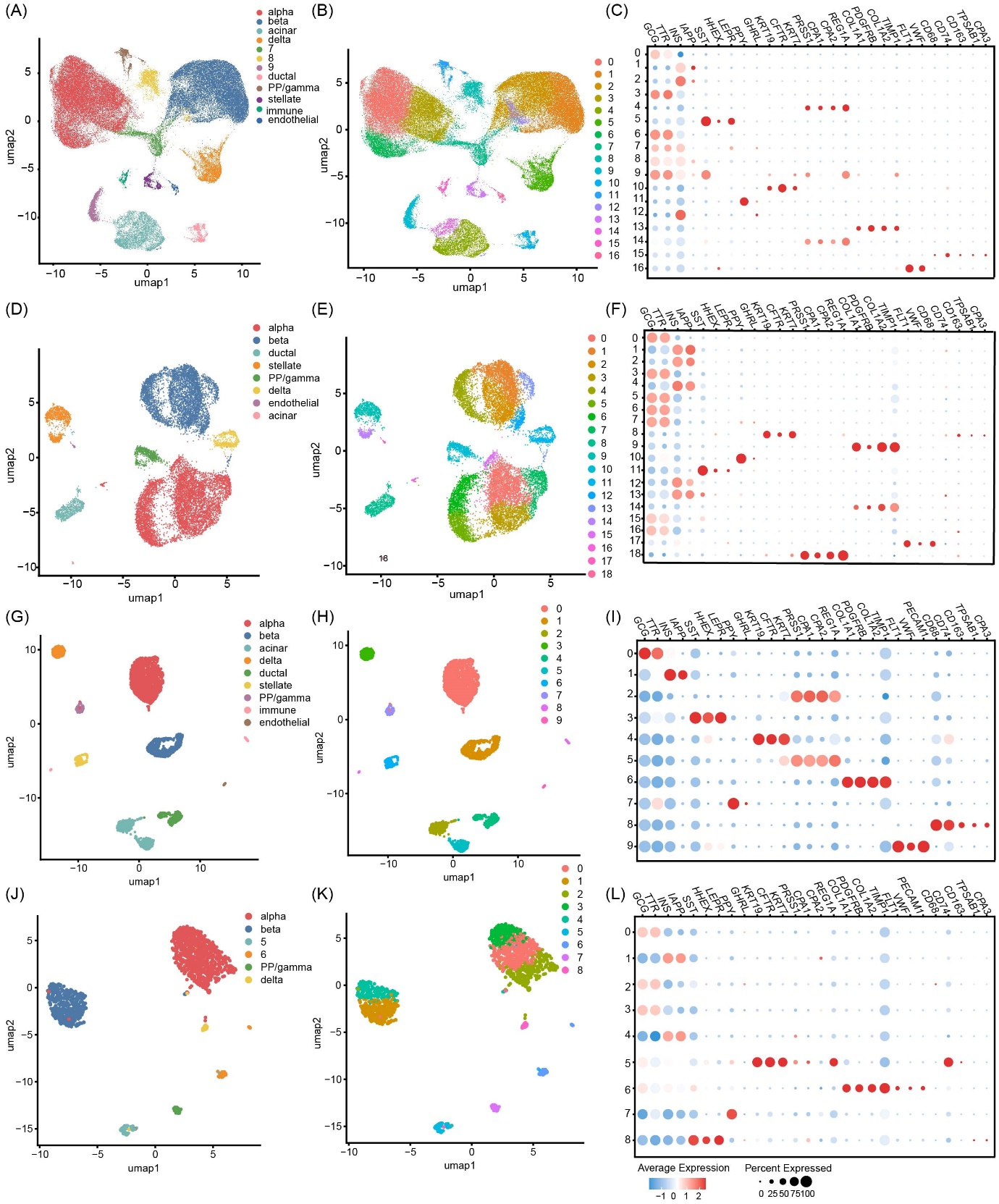


### Figure S7. Data preprocessing in four validation datasets for scRiskCell.

(A–L) Cell clustering and annotation for different validation datasets, for validation dataset 1 (A–C: *n* = 36,923 beta cells), validation dataset 2 (D–F: *n* = 5,402 beta cells), validation dataset 3 (G–I: *n* = 537 beta cells), and validation dataset 4 (J–L: *n* = 419 beta cells).


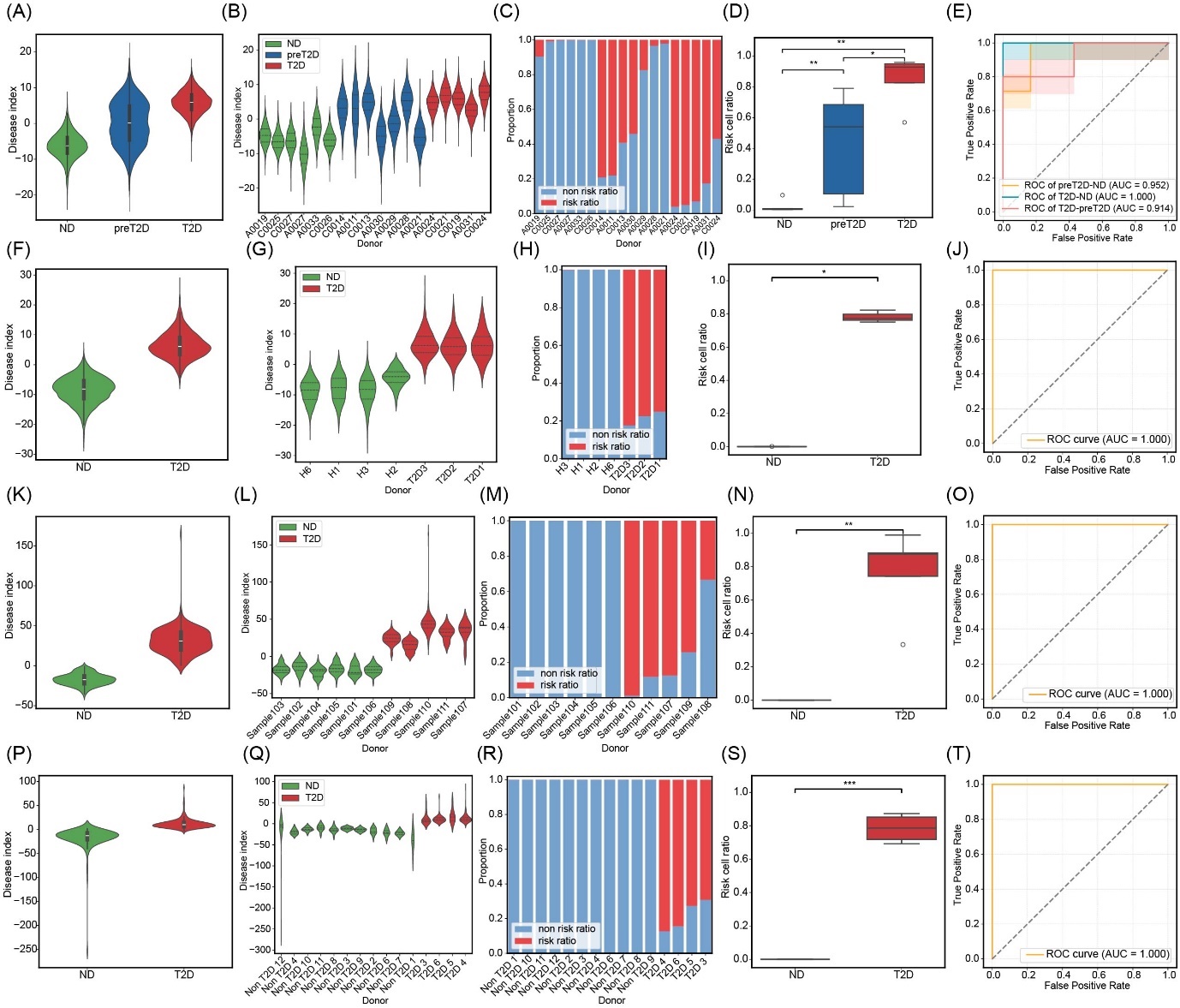


### Figure S8. scRiskCell identifies type 2 diabetes (T2D)-associated beta risk cells in validation datasets.

(A–T) scRiskCell was applied to beta cells in four different validation datasets: dataset 1 (A–E, ND *n* = 6, preT2D *n* = 7, T2D *n* = 5, *p*-values in D were derived from two-sided Wilcoxon rank-sum tests and adjusted using the Benjamini-Hochberg (BH) method; Significance levels: * *p*.adj < 0.05, ** *p*.adj < 0.01, *** *p*.adj < 0.001); dataset 2 (F–J, ND *n* = 4 donors, T2D *n* = 3 donors, two-sided Wilcoxon rank-sum test. Significance levels: * *p*-value < 0.05, ** *p*-value < 0.01, *** *p*-value < 0.001.); dataset 3 (K–O, ND *n* = 6 donors, T2D *n* = 5 donors, two-sided Wilcoxon rank-sum test. Significance levels: * *p*-value < 0.05, ** *p*-value < 0.01, *** *p*-value < 0.001.); dataset 4 (P–T: ND *n* = 11 donors, T2D *n* = 4 donors; two-sided Wilcoxon rank-sum test. Significance levels: * *p*-value < 0.05, ** *p*-value < 0.01, *** *p*-value < 0.001.)


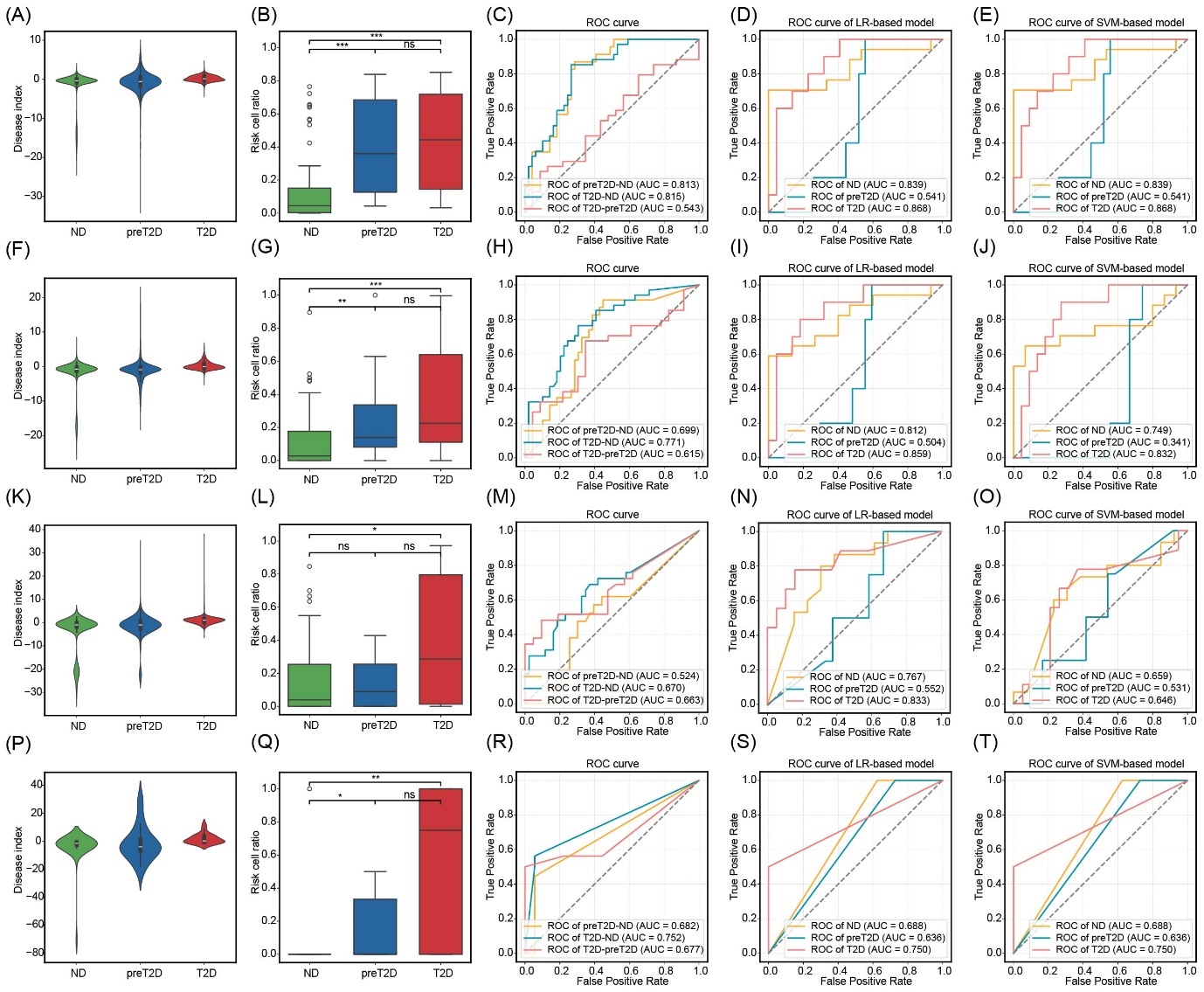


### Figure S9. scRiskCell identifies cell-type-specific risk cells associated with type 2 diabetes (T2D).

(A–T) scRiskCell was applied to different cell types, to alpha cells (A–E: ND *n* = 49 donors; preT2D *n* = 23 donors; T2D *n* = 34 donors); to delta cells (F–J: ND *n* = 49 donors; preT2D *n* = 23 donors; T2D *n* = 34 donors); to gamma cells (K–O: ND *n* = 43 donors; preT2D *n* = 21 donors; T2D *n* = 29 donors); and to epsilon cells (P–T: ND *n* = 18 donors; preT2D *n* = 9 donors; T2D *n* = 16 donors). Box plots showing individual risk cell ratio in three clinical groups. The box plot center line, limits and whiskers represent the median, quartiles and 1.5× interquartile range, respectively. Two-sided Wilcoxon rank-sum test. *P*-values were adjusted for multiple comparisons using the Benjamini-Hochberg (BH) method. Significance levels: ns *p*.adj ≥ 0.05, * *p*.adj < 0.05, ** *p*.adj < 0.01, *** *p*.adj < 0.001.


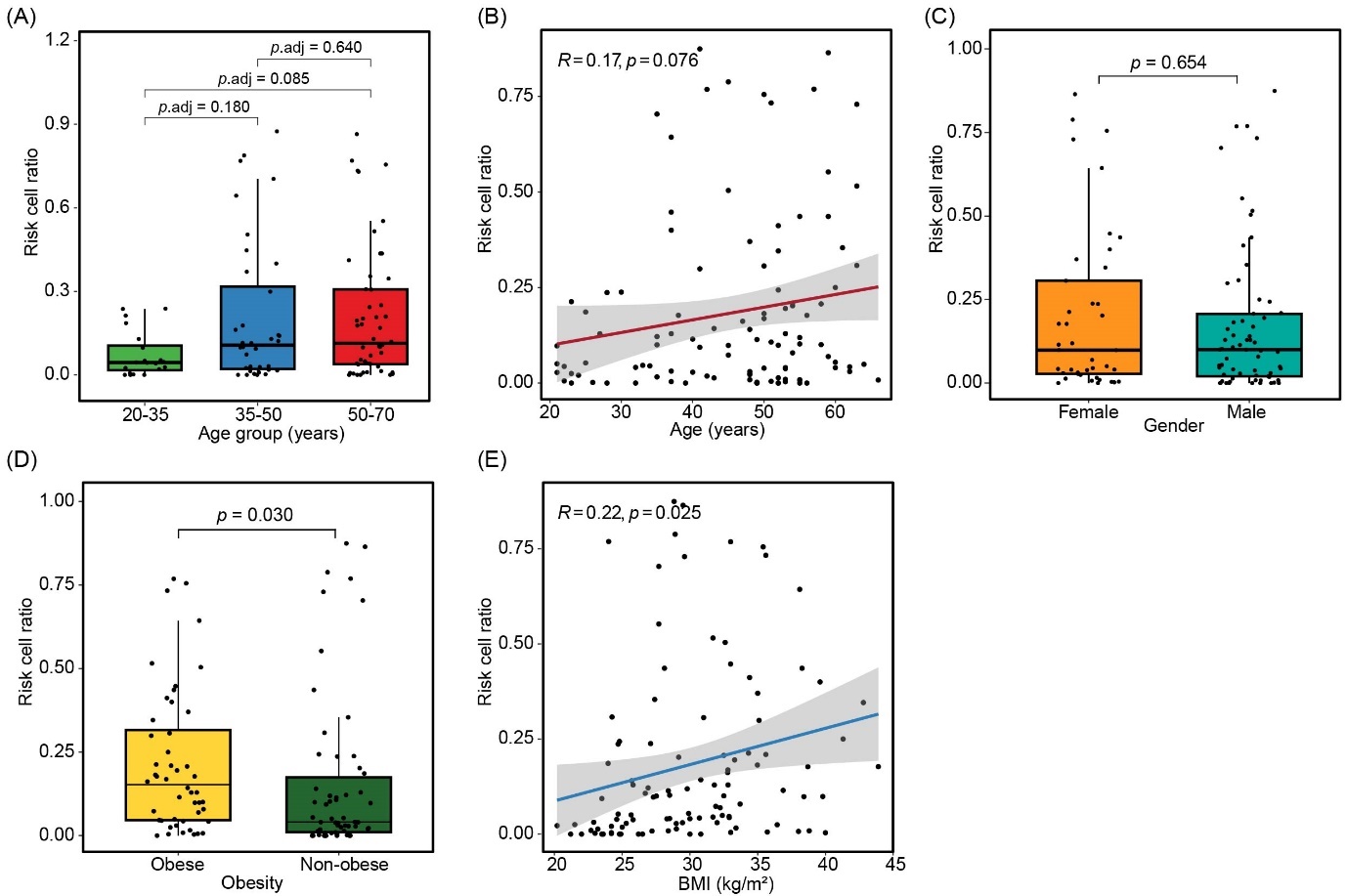


### Figure S10. Risk beta cell proportion mirrors clinical traits.

(A) Beta risk cell ratio comparisons of different age group. Dots denote data points from individual donors, with [20, 35) *n* = 20 donors; [35，50) *n* = 36 donors; [50, 70) *n* = 50 donors. Two-sided Wilcoxon rank-sum test. *P*-values were adjusted for multiple comparisons using the Benjamini-Hochberg (BH) method. (B) Pearson correlation between age and beta risk cell ratio across donors (*n* = 106 donors). Pearson correlation was assessed using a two-sided *t*-test for the correlation coefficient. The bands around the linear regression line represent the range of 95% confidence interval of the risk cell ratio. (C) Beta risk cell ratio of different gender group. Female *n* = 41; male *n* = 65. *P*-value was calculated using a two-sided Wilcoxon rank-sum test. (D) Beta risk cell ratio of different obesity group. Obese *n* = 48; Non-obese *n* = 58. Two-sided Wilcoxon rank-sum test. (E) Pearson correlation between BMI and beta risk cell ratio across donors (*n* = 106 donors). Pearson correlation was assessed using a two-sided *t*-test for the correlation coefficient. The bands around the linear regression line represent the range of 95% confidence interval of the risk cell ratio.


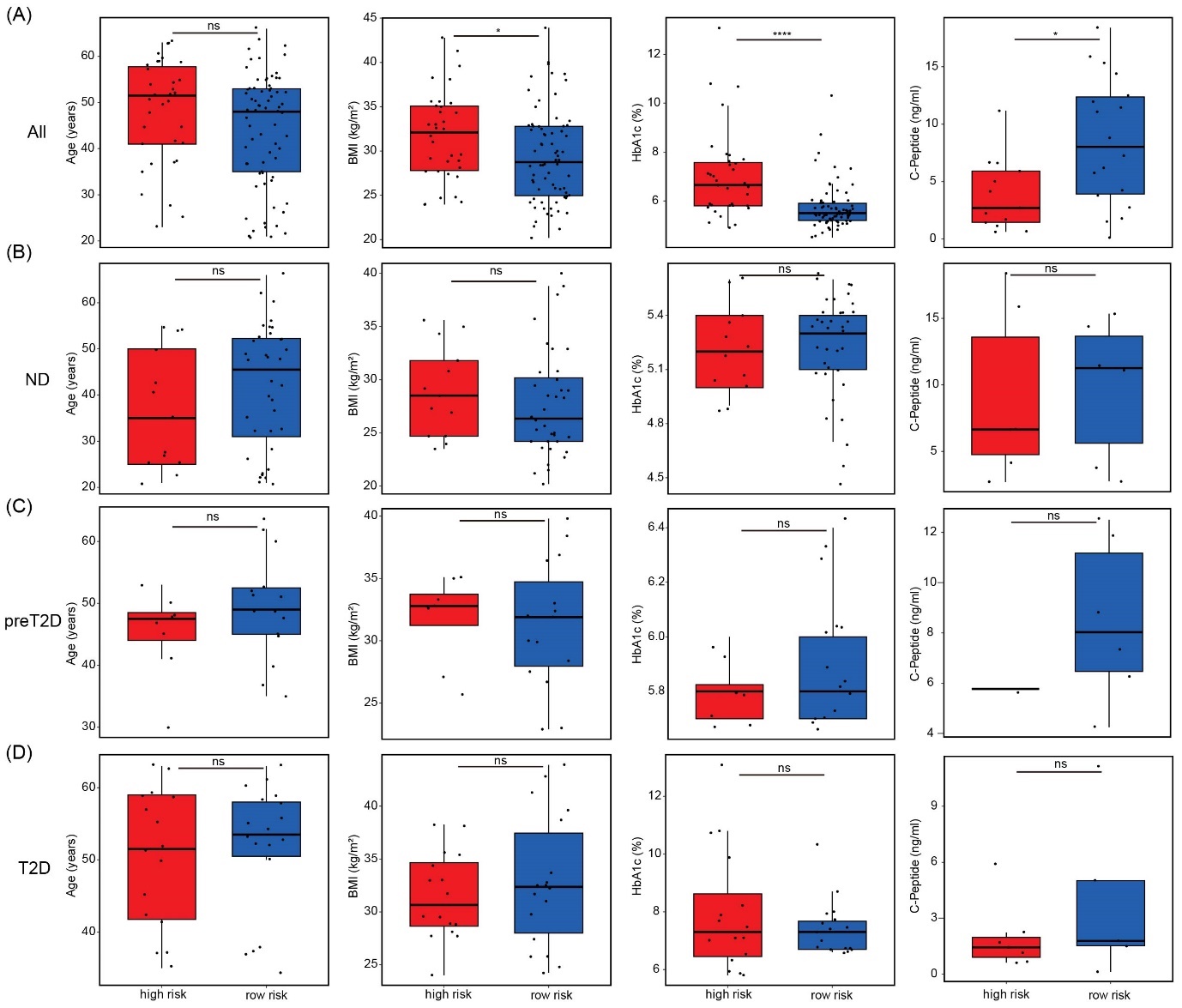


### Figure S11. Comparison of clinical features within disease and disease subgroups.

(A–D) Comparison of clinical features within the high-low risk group in all samples (A: age, BMI and HbA1c: high risk *n* = 34, low risk *n* = 72; C-peptide: high risk *n* = 13, low risk *n* = 18); in ND samples (B: age, BMI: high risk *n* = 13, low risk *n* = 36; HbA1c: high risk *n* = 12, low risk *n* = 36; C-peptide: high risk *n* = 6, low risk *n* = 6); in preT2D samples (C: age, BMI and HbA1c: high risk *n* = 8, low risk *n* = 15; C-peptide: high risk *n* = 1, low risk *n* = 6); and in T2D samples (D: age, BMI and HbA1c: high risk *n* = 16, low risk *n* = 18; C-peptide: high risk *n* = 7, low risk *n* = 5). Dots denote data points from individual donors. Two-sided Wilcoxon rank-sum test. Significance levels: ns *p*-value ≥ 0.05, * *p*-value < 0.05, ** *p*-value < 0.01, *** *p*-value < 0.001, **** *p*-value < 0.0001.


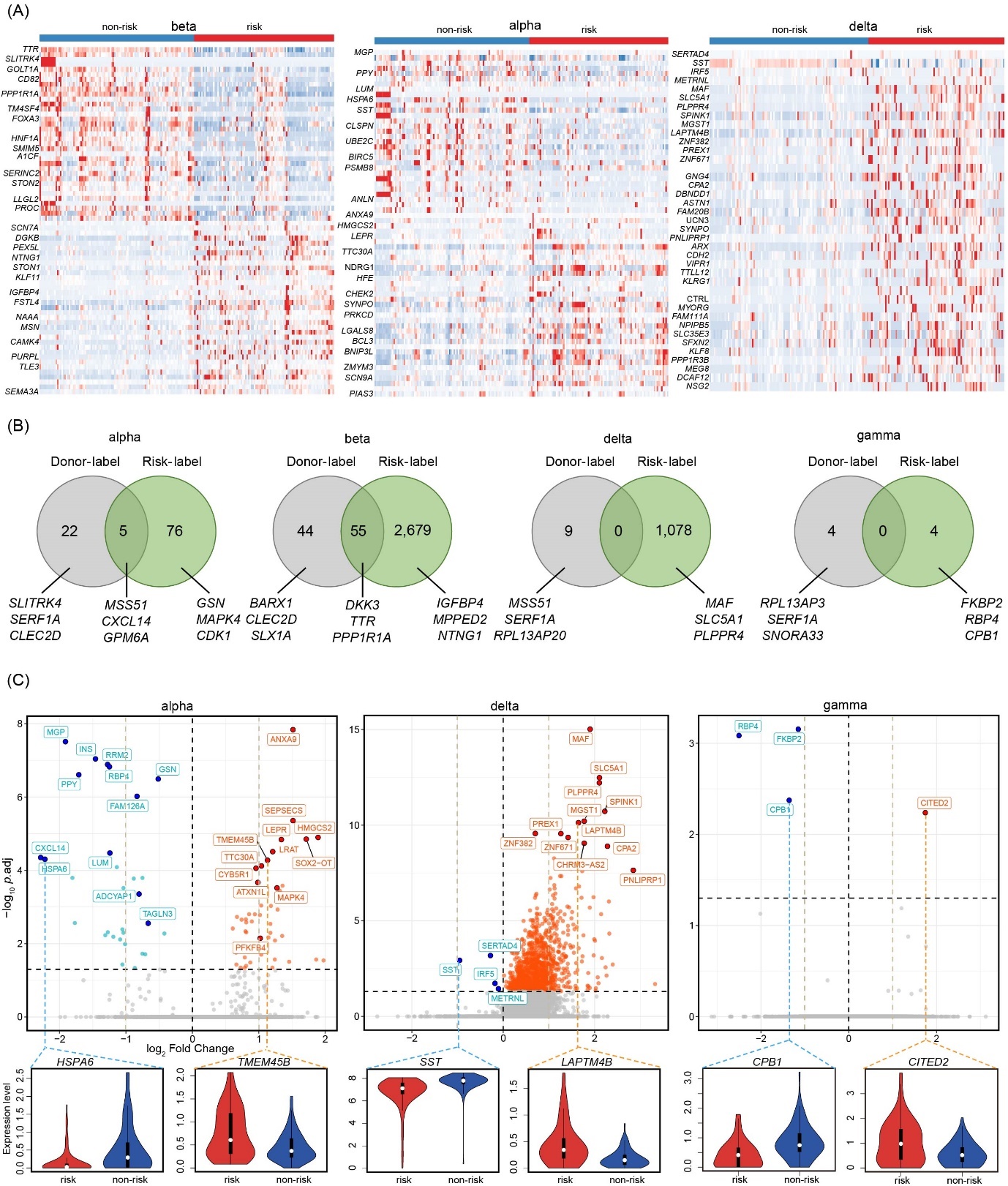


### Figure S12. Molecular specific changes associated with risk cells.

(A) Heatmap showing top significantly differentially expressed genes (*p*.adj < 0.05) between risk and non-risk cells in beta, alpha, and delta cells. Adjusted *p*-values were derived from pseudobulk differential expression analysis using DESeq2 with Benjamini-Hochberg (BH) correction. (B) Venn diagrams illustrating the shared and distinct DEGs identified through conventional donor label-based (ND vs T2D) analysis and risk cell-based analysis across different cell types. Representative genes for each category are listed below the corresponding diagram. (C) Gene with significantly differential expression changes at the pseudobulk (sample) level using DESeq2 between risk and non-risk cells of alpha/deta/gamma cells. Genes with an adjusted *p*-value < 0.05 were considered statistically differentially expressed. (Bottom) Violin plots highlight examples of differentially expressed genes with different dysregulation directions in each cell type.


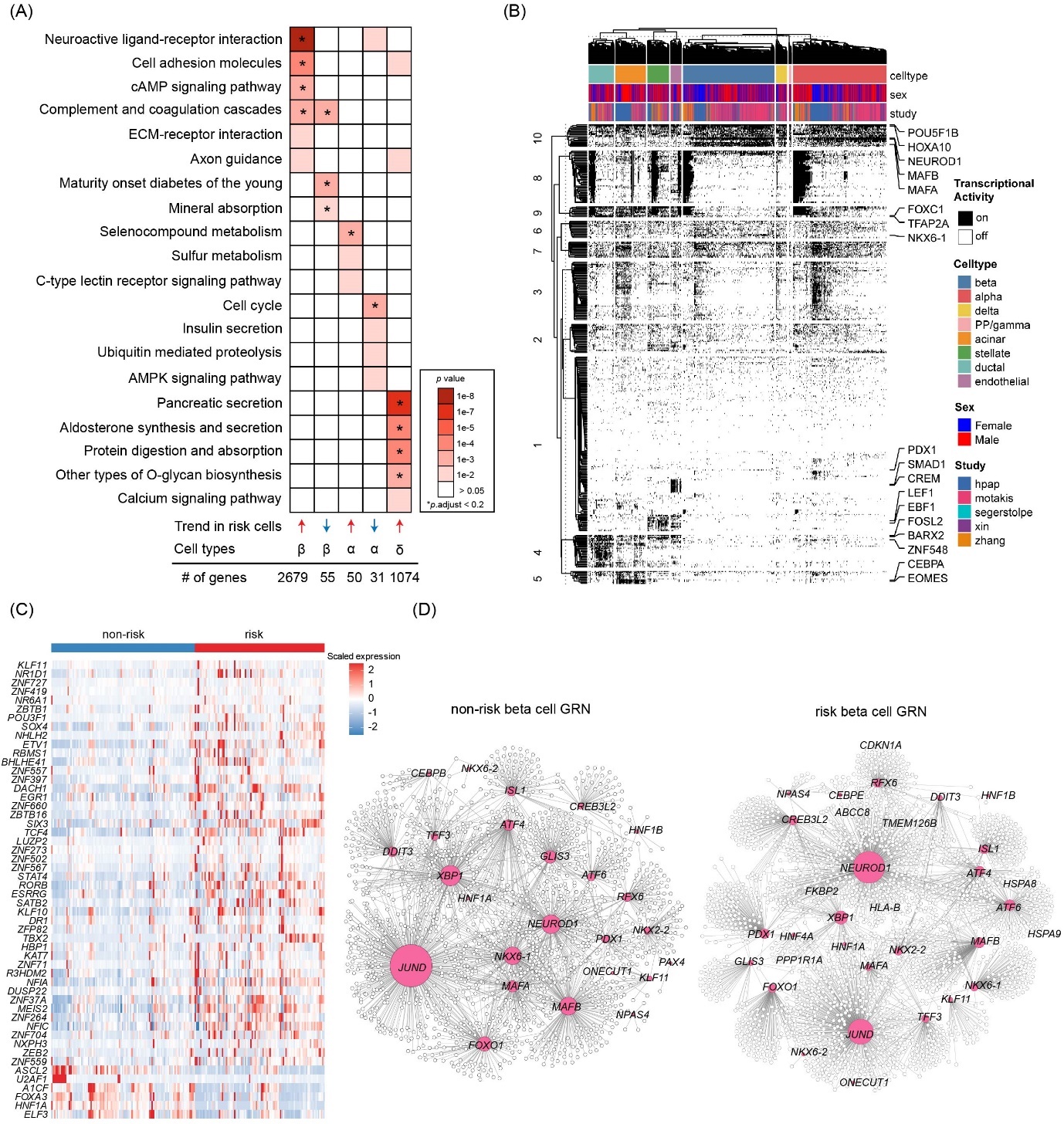


### Figure S13. Gene regulatory networks changes associated with risk cells.

(A) Enrichment analysis for DEGs based on risk cells. Total number of genes of each function term is shown below. (B) Heatmap and hierarchical clustering analysis of TF activity patterns. TFs classified as “on” are shown in black, while those classified as “off” are displayed in white. The top rows of the heatmap indicate the distribution of cells, categorized by cell type, sex, and study origin. The column dendrogram represents hierarchical clustering clades within each cell type, using Euclidean distances. (C) Heatmap showing top significantly differentially expressed TFs between beta risk and non-risk cells. Relative gene expression is shown in pseudo color. TF genes with an adjusted *p*-value < 0.05 were considered statistically differentially expressed. (D) Gene regulatory networks (GRNs) formed by TFs and its targets identified using SCENIC in beta non-risk (left) and risk (right) cells, respectively. TFs are shown as pink nodes, and target protein-coding genes are depicted in white. Gray lines connecting the nodes represent the importance metric between TF-gene pairs, where thicker edges indicate stronger TF-target relationship. Node size represents the “degree” measurements that represent the number of target genes of a given TF within the network.


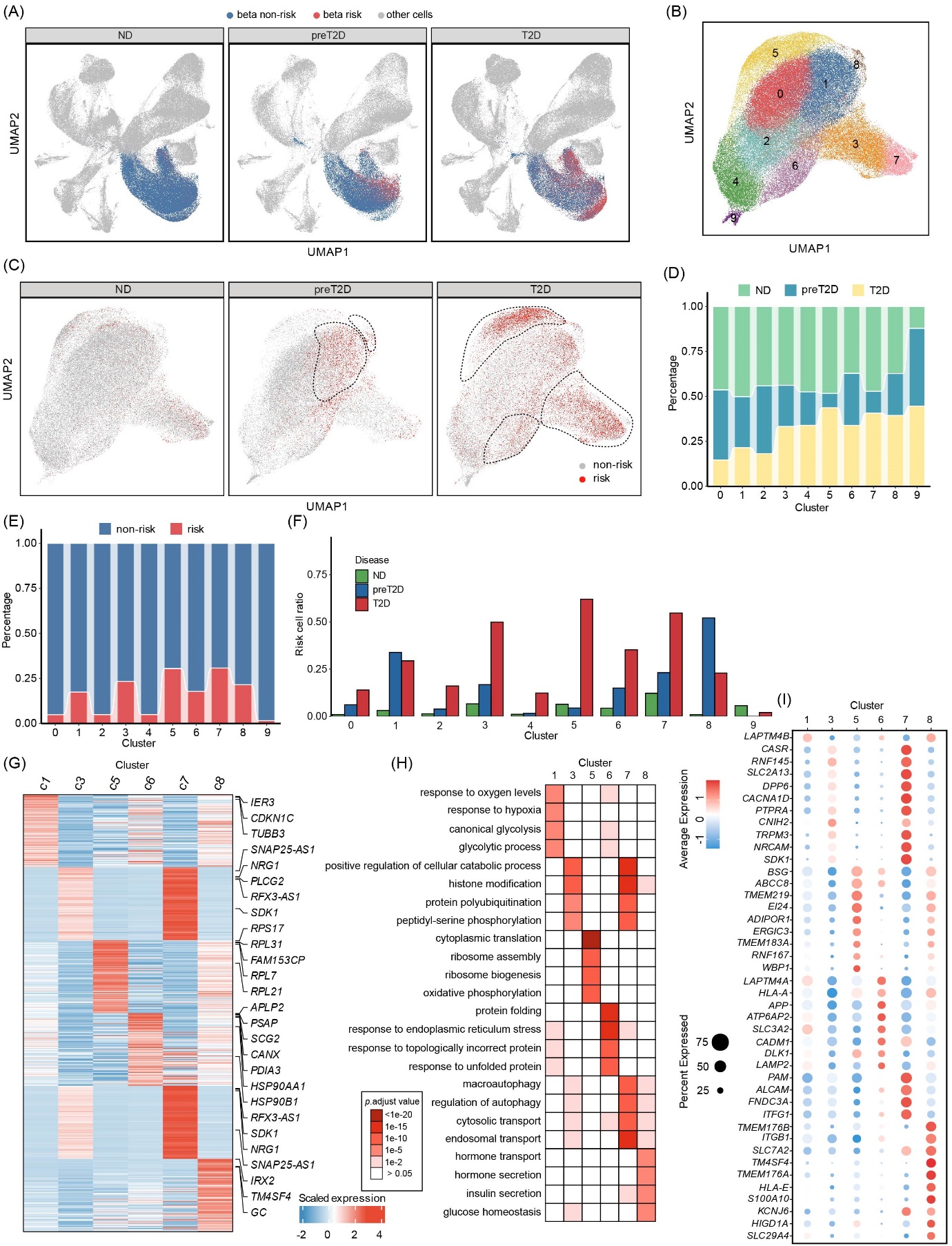


### Figure S14. Risk cell aggregation patterns of beta cell subsets in diabetes progression.

(A) Projection of all cells (*n* = 495,945) from ND (cells: *n* = 208,753), preT2D (cells: *n* = 112,938) and T2D donors (cells: *n* = 174,254) using UMAP analysis. All cells except beta cells are shown in gray. (B) Projection of beta cells (*n* = 144,172) from ND (cells: *n* = 65,634), preT2D (cells: *n* = 39,832) and T2D donors (cells: *n* = 38,706) using UMAP analysis. (C) Aggregation patterns of risk beta cells in different disease states. (D) Bar graphs demonstrating the proportion of different diseased cells (cells from different clinical groups) in each beta cell subpopulations. (E) Stacked bar plots showing the proportion of beta risk and non-risk cells in each beta cell subgroups. (F) Bar chart shows the proportion of risk cells in each beta cell subsets across three disease states. (G) Heatmap showing top significantly differentially expressed genes across different beta subpopulations. Genes with an adjusted *p*-value < 0.05 were considered statistically differentially expressed. Adjusted *p*-values were derived from pseudobulk differential expression analysis using DESeq2 with Benjamini-Hochberg (BH) correction. Relative gene expression is shown in pseudo color. Values represent average expression after mean-centering and scaling (*z*-score) across cell types. Negative values indicate expression below the gene's average across cell types. (H) Enrichment analysis for enriched genes of each beta subclusters. Cells are colored according to *p*.adj. (I) Bubble plot showing the expression patterns of top endocrine surface genes.


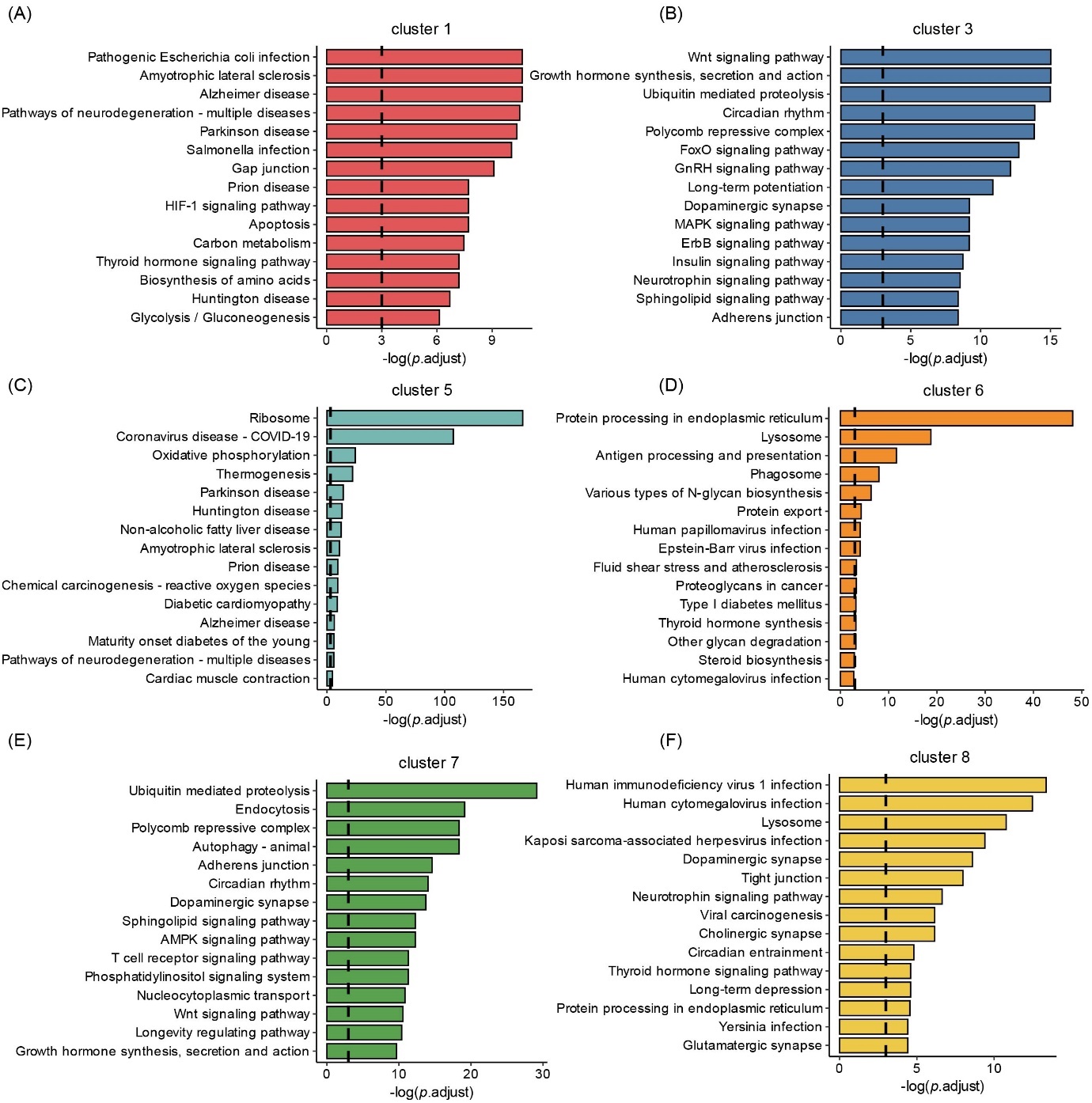


### Figure S15. Enrichment analysis of beta subpopulation markers.

(A–F) Pathway enrichment analysis of DEGs in Cluster 1, 3, 5, 6, 7, and 8 beta cells ordered by statistical significance. A *p*.adj < 0.05 was considered statistically significant.

### Reference

1. Lawlor, Nathan, Joshy George, Mohan Bolisetty, Romy Kursawe, Lili Sun, V Sivakamasundari, Ina Kycia, Paul Robson, Michael L Stitzel. 2017. “Single-cell transcriptomes identify human islet cell signatures and reveal cell-type–specific expression changes in type 2 diabetes.” *Genome research* 27: 208–222. https://doi.org/10.1101/gr.212720.116

2. Collombat, Patrick, Jacob Hecksher-Sørensen, Jens Krull, Joachim Berger, Dietmar Riedel, Pedro L Herrera, Palle Serup, Ahmed Mansouri. 2007. “Embryonic endocrine pancreas and mature β cells acquire α and PP cell phenotypes upon Arx misexpression.” *The Journal of clinical investigation* 117: 961–970. https://doi.org/10.1172/JCI29115

3. Wang, Gaowei, Joshua Chiou, Chun Zeng, Michael Miller, Ileana Matta, Jee Yun Han, Nikita Kadakia, et al. 2023. “Integrating genetics with single-cell multiomic measurements across disease states identifies mechanisms of beta cell dysfunction in type 2 diabetes.” *Nature Genetics* 55: 984–994. https://doi.org/10.1038/s41588-023-01397-9

4. Marselli, Lorella, Emanuele Bosi, Carmela De Luca, Silvia Del Guerra, Marta Tesi, Mara Suleiman, Piero Marchetti. 2021. “Arginase 2 and polyamines in human pancreatic beta cells: Possible role in the pathogenesis of type 2 diabetes.” *International Journal of Molecular Sciences* 22: 12099. https://doi.org/10.3390/ijms222212099

5. Taneera, Jalal, Abdul Khader Mohammed, Anila Khalique, Bashair M. Mussa, Nabil Sulaiman, Yasser Bustanji, Mohamed A. Saleh, Mohamed Madkour, Eman Abu-Gharbieh, Waseem El-Huneidi. 2024. “Unraveling the significance of PPP1R1A gene in pancreatic β-cell function: A study in INS-1 cells and human pancreatic islets.” *Life Sciences* 345: 122608. https://doi.org/10.1016/j.lfs.2024.122608

6. Cobb, Melanie H, Jessie E Hepler, Mangeng Cheng, David Robbins.1994. The mitogen-activated protein kinases, ERK1 and ERK2. *Seminars in cancer biology*;5:261–268.

7. Weng, Chen, Anniya Gu, Shanshan Zhang, Leina Lu, Luxin Ke, Peidong Gao, Xiaoxiao Liu, et al. 2023. “Single cell multiomic analysis reveals diabetes-associated β-cell heterogeneity driven by HNF1A.” *Nature Communications* 14: 5400. https://doi.org/10.1038/s41467-023-41228-3

8. Fang, Zhou, Chen Weng, Haiyan Li, Ran Tao, Weihua Mai, Xiaoxiao Liu, Leina Lu, et al. 2019. “Single-cell heterogeneity analysis and CRISPR screen identify key β-cell-specific disease genes.” *Cell Reports* 26: 3132–3144. https://doi.org/10.1016/j.celrep.2019.02.043

9. Ng, Natasha Hui Jin, Soumita Ghosh, Chek Mei Bok, Carmen Ching, Blaise Su Jun Low, Juin Ting Chen, Euodia Lim, et al. 2024. “HNF4A and HNF1A exhibit tissue specific target gene regulation in pancreatic beta cells and hepatocytes.” *Nature Communications* 15: 4288. https://doi.org/10.1038/s41467-024-48647-w

10. Boesgaard, T. W., N. Grarup, T. Jørgensen, K. Borch-Johnsen, T. Hansen, O. Pedersen, Glucose Meta-Analysis of, Consortium Insulin-Related Trait. 2010. “Variants at DGKB/TMEM195, ADRA2A, GLIS3 and C2CD4B loci are associated with reduced glucose-stimulated beta cell function in middle-aged Danish people.” *Diabetologia* 53: 1647–1655. https://doi.org/10.1007/s00125-010-1753-5

11. Rung, Johan, Stéphane Cauchi, Anders Albrechtsen, Lishuang Shen, Ghislain Rocheleau, Christine Cavalcanti-Proença, François Bacot, et al. 2009. “Genetic variant near IRS1 is associated with type 2 diabetes, insulin resistance and hyperinsulinemia.” *Nature Genetics* 41: 1110–1115. https://doi.org/10.1038/ng.443

12. Segerstolpe, Åsa, Athanasia Palasantza, Pernilla Eliasson, Eva-Marie Andersson, Anne-Christine Andréasson, Xiaoyan Sun, Simone Picelli, et al. 2016. “Single-cell transcriptome profiling of human pancreatic islets in health and type 2 diabetes.” *Cell Metabolism* 24: 593–607. https://doi.org/10.1016/j.cmet.2016.08.020

13. Li, Guoqiang, Lijun Zhu, Mingwei Guo, Dongmei Wang, Meiyao Meng, Yinzhao Zhong, Zhijian Zhang, et al. 2023. “Characterisation of forkhead box protein A3 as a key transcription factor for hepatocyte regeneration.” *JHEP Reports* 5: 100906. https://doi.org/10.1016/j.jhepr.2023.100906

14. Vieira, Elaine, Laura Marroquí, Ana Lucia C Figueroa, Beatriz Merino, Rebeca Fernandez-Ruiz, Angel Nadal, Thomas P Burris, Ramon Gomis, Ivan Quesada. 2013. “Involvement of the clock gene Rev-erb alpha in the regulation of glucagon secretion in pancreatic alpha-cells.” *PloS one* 8: e69939. https://doi.org/10.1371/journal.pone.0069939

15. Van de Sande, Bram, Christopher Flerin, Kristofer Davie, Maxime De Waegeneer, Gert Hulselmans, Sara Aibar, Ruth Seurinck, et al. 2020. “A scalable SCENIC workflow for single-cell gene regulatory network analysis.” *Nature Protocols* 15: 2247–2276. https://doi.org/10.1038/s41596-020-0336-2

16. Shrestha, Shristi, Galina Erikson, James Lyon, Aliya F Spigelman, Austin Bautista, Jocelyn E Manning Fox, Cristiane Dos Santos, Maxim Shokhirev, Jean-Philippe Cartailler, Martin W Hetzer. 2022. “Aging compromises human islet beta cell function and identity by decreasing transcription factor activity and inducing ER stress.” *Science Advances* 8: eabo3932. https://doi.org/10.1126/sciadv.abo3932

17. Yu, Junqin, Jianlong Ma, Yanfeng Li, Yang Zhou, Lingfei Luo, Yun Yang. 2023. “Pax4-ghrelin mediates the conversion of pancreatic ε-cells to β-cells after extreme β-cell loss in zebrafish.” *Development* 150: dev201306. https://doi.org/10.1242/dev.201306

18. Kordowich, Simon, Ahmed Mansouri, Patrick Collombat. 2010. “Reprogramming into pancreatic endocrine cells based on developmental cues.” *Molecular and Cellular Endocrinology* 323: 62–69. https://doi.org/10.1016/j.mce.2009.12.016

19. Wang, Jing, Xin Yang, Jingjing Zhang. 2016. “Bridges between mitochondrial oxidative stress, ER stress and mTOR signaling in pancreatic β cells.” *Cellular Signalling* 28: 1099–1104. https://doi.org/10.1016/j.cellsig.2016.05.007

20. Fonseca, Sonya G., Jesper Gromada, Fumihiko Urano. 2011. “Endoplasmic reticulum stress and pancreatic β-cell death.” *Trends in Endocrinology & Metabolism* 22: 266–274. https://doi.org/10.1016/j.tem.2011.02.008

21. Johnston, Natalie R, Ryan K Mitchell, Elizabeth Haythorne, Maria Paiva Pessoa, Francesca Semplici, Jorge Ferrer, Lorenzo Piemonti, et al. 2016. “Beta cell hubs dictate pancreatic islet responses to glucose.” *Cell Metabolism* 24: 389–401. https://doi.org/10.1016/j.cmet.2016.06.020

22. Xie, Xueqin, Changchun Wu, Yuduo Hao, Tianyu Wang, Yuhe Yang, Peiling Cai, Yang Zhang, et al. 2023. “Benefits and risks of drug combination therapy for diabetes mellitus and its complications: a comprehensive review.” *Frontiers in Endocrinology* 14: 1301093. https://doi.org/10.3389/fendo.2023.1301093

23. Shapira, Suzanne N., Ali Naji, Mark A. Atkinson, Alvin C. Powers, Klaus H. Kaestner. 2022. “Understanding islet dysfunction in type 2 diabetes through multidimensional pancreatic phenotyping: The human pancreas analysis program.” *Cell Metabolism* 34: 1906–1913. https://doi.org/10.1016/j.cmet.2022.09.013

24. Xin, Yurong, Giselle Dominguez Gutierrez, Haruka Okamoto, Jinrang Kim, Ann-Hwee Lee, Christina Adler, Min Ni, George D. Yancopoulos, Andrew J. Murphy, Jesper Gromada. 2018. “Pseudotime ordering of single human β-cells reveals states of insulin production and unfolded protein response.” *Diabetes* 67: 1783–1794. https://doi.org/10.2337/db18-0365

25. Xin, Yurong, Jinrang Kim, Haruka Okamoto, Min Ni, Yi Wei, Christina Adler, Andrew J Murphy, George D Yancopoulos, Calvin Lin, Jesper Gromada. 2016. “RNA sequencing of single human islet cells reveals type 2 diabetes genes.” *Cell Metabolism* 24: 608–615. https://doi.org/10.1016/j.cmet.2016.08.018

26. Ngara, Mtakai, Nils Wierup. 2022. “Lessons from single-cell RNA sequencing of human islets.” *Diabetologia* 65: 1241–1250. https://doi.org/10.1007/s00125-022-05699-1

27. Chiou, J., C. Zeng, Z. Cheng, J. Y. Han, M. Schlichting, M. Miller, R. Mendez, et al. 2021. “Single-cell chromatin accessibility identifies pancreatic islet cell type- and state-specific regulatory programs of diabetes risk.” *Nature Genetics* 53: 455-466. https://doi.org/10.1038/s41588-021-00823-0

28. Shrestha, S., D. C. Saunders, J. T. Walker, J. Camunas-Soler, X. Q. Dai, R. Haliyur, R. Aramandla, et al. 2021. “Combinatorial transcription factor profiles predict mature and functional human islet alpha and beta cells.” *JCI Insight* 6: https://doi.org/10.1172/jci.insight.151621

29. Da Cunha, JPC, PAF Galante, JE De Souza, RF De Souza, PM Carvalho, DT Ohara, RP Moura, SM Oba-Shinja, Suely Kazue Nagahashi Marie, WA Silva Jr. 2009. “Bioinformatics construction of the human cell surfaceome.” *Proceedings of the National Academy of Sciences* 106: 16752–16757. https://doi.org/10.1073/pnas.0907939106

30. Squair, Jordan W., Matthieu Gautier, Claudia Kathe, Mark A. Anderson, Nicholas D. James, Thomas H. Hutson, Rémi Hudelle, et al. 2021. “Confronting false discoveries in single-cell differential expression.” *Nature Communications* 12: 5692. https://doi.org/10.1038/s41467-021-25960-2

31. Wu, Tianzhi, Erqiang Hu, Shuangbin Xu, Meijun Chen, Pingfan Guo, Zehan Dai, Tingze Feng, et al. 2021. “clusterProfiler 4.0: A universal enrichment tool for interpreting omics data.” *The Innovation* 2: 100141. https://doi.org/10.1016/j.xinn.2021.100141

32. Aibar, Sara, Carmen Bravo González-Blas, Thomas Moerman, Vân Anh Huynh-Thu, Hana Imrichova, Gert Hulselmans, Florian Rambow, et al. 2017. “SCENIC: Single-cell regulatory network inference and clustering.” *Nature Methods* 14: 1083–1086. https://doi.org/10.1038/nmeth.4463
